# Shen-Ling-Bai-Zhu-San (SL) and SL Derived-Polysaccharide (PL) Ameliorate the Severity of Diarrhea-Induced by High Lactose via Modification of Colonic Fermentation

**DOI:** 10.3389/fphar.2022.883355

**Published:** 2022-06-28

**Authors:** Hong Xue, Jinxin Ma, Yitian Wang, Mengxiong Lu, Fengyun Wang, Xudong Tang

**Affiliations:** ^1^ Digestive Laboratory of Traditional Chinese Medicine Research Institute of Spleen and Stomach Diseases, Xiyuan Hospital, China Academy of Chinese Medical Sciences, Beijing, China; ^2^ Department of Integrated Traditional Chinese and Western Medicine, Peking University Health Science Center, Beijing, China; ^3^ Department of Gastrointestinal Medicine, Peking University Traditional Chinese Medicine Clinical Medican School (Xiyuan), Beijing, China

**Keywords:** shen-ling-bai-zhu-san (SL), polysaccharide (PL), microbiota, fermentation, ion transporter, lactose-induced diarrhea

## Abstract

In our previous study, we demonstrated that Shen-ling-bai-zhu-san (SL), a classical Chinese herbal formula, could alleviate lactose-induced diarrhea. However, little is known about the mechanism underlying SL action or the efficacy of the polysaccharide (PL) derived from SL. In this study, we investigated the effect of SL and PL on improving the dysregulated luminal and mucosal microbiota in rats with high lactose diet using 16S rRNA analysis. The concentrations of lactose, lactic acid in cecum and short-chain fatty acids (SCFAs) in cecum and portal vein were measured, meanwhile the expression of ion transporters were ascertained. Our data suggest that the SL, PL and cecal microbiota transplantation (CMT) significantly decreased fecal water content and water intake. In the luminal microbiota there was a significant increase in Akkermansia, Bifidobacterium and Blautia and a lower abundance of *Lactobacillus*, Escherichia-Shigella, and Dubosiella, while the mucosal microbiota showed a significant increase in Bifidobacterium, Akkermansia, Albaculum, Bilophila, and Coriobacteriaceae_UCG-002 and a lower abundance of *Enterococcus*, *Helicobacter*, Dubosiella, and Collinsella. Furthermore, the treatments enhanced lactose fermentation and SCFA production, which may be related to the modulation of the luminal microbial community. A lower ratio of phosphorylation Na/H exchanger3/Na/H exchanger3 (pNHE3/NHE3) and a higher sodium monocarboxylate1 (sMCT1) expression were found in the treatment group than in the model group, which may be related to the changes in the mucosal microbial community. Also, the treatments may restore the impacted metabolic pathways of gut microbiota. These results provide an important foundation for mechanism of SL action and developing PL-based treatment for lactose-induced diarrhea.

## Introduction

Diarrhea is a highly prevalent and bothersome disorder and a severe worldwide public health issue, especially in developing countries (Thapar and Sanderson, 2004; Zangenberg et al., 2019). Lactose malabsorption or intolerance affects 20% of the world population and is frequently associated with diarrheal conditions (Arasaradnam et al., 2018; Hiner and Walters, 2021). Lactose malabsorption (LM) refers to any cause of a failure to digest and/or absorb lactose in the small intestine (Misselwitz et al., 2019). Lactose intolerance (LI) is the occurrence of symptoms such as abdominal pain, bloating or diarrhea in LM patients following their ingestion of lactose (Misselwitz et al., 2019). Undigested lactose in the small intestine leads to an osmotic trapping of water, which increases the colon’s osmotic load. Lactose is first hydrolyzed to glucose and galactose, catalyzed by the enzyme β-galactosidase. Glucose and galactose are subsequently fermented by gut microbiota, resulting in the production of the intermediate such as lactate and end-product metabolites (short-chain fatty acids, SCFAs) (He et al., 2006; Windey et al., 2015; Misselwitz et al., 2019). Diarrhea occurs if the load of lactose exceeds the fermentation capacity of the colon or if the SCFA load exceeds the colon’s capacity for absorption (Binder, 2010). Under typical physiological conditions (intestinal lumen pH 5–7), SCFAs are specifically transported by monocarboxylate (MCT1) and sodium MCT1 (sMCT1) transporters that are present in the colonocyte brush border membranes (Sivaprakasam et al., 2017). Once absorbed, SCFAs are carried in the portal vein to the liver, where they are oxidized. The colonic epithelium allows for the efficient salvage of water and solutes (Na^+^, Cl^−^, SCFAs) and the maintenance of the resident microbial ecosystem. It is generally accepted that electroneutral NaCl absorption by the intestine largely reflects the tandem operation of apical Na/H exchange and Cl/HCO3 exchange processes, primarily involving the Na/H exchanger3 (NHE3) and the Cl/HCO3 exchanger solute carrier family 26 member 3(SLC26A3) (Gill et al., 2003; Pradeep et al., 2003).

The colon is inhabited by a wide variety of bacteria, archaea, and fungi, and there are approximately 10^14^ microorganisms in the gut, encoding more than three million genotypes (Lozupone et al., 2012). The colonic microbiota, which ferments lactose, is a key factor in the colonic processing of lactose. Our recent study demonstrated that there are amounts of lactose and lactate but little SCFA in the cecal content associated with the depletion of the Lachnospiraceae NK4A136 group and Ruminococcaceae UCG-005 and a concurrent increase in the relative abundance of *Lactobacillus*, Escherichia-Shigella and Megamonas in the cecal microbiota of rats with high lactose diet (HLD) (Xue et al., 2020). Since it is commonly believed that lactose intolerance causes diarrhea, milk and milk products are often reduced or eliminated from the diet of patients with diarrheal disorders (Fassio et al., 2018). It is of some importance to understand whether such dietary manipulation is truly beneficial since milk is a major natural dietary source of calcium and an inexpensive source of protein. Few treatments are available, and many lactose-intolerant people are left with no other choice than to practice an exclusion diet that could lead to mineral and vitamin deficiencies.

Traditional Chinese medicine (TCM) has been used for several millennia to treat diarrheal diseases in China and other developing countries, and now, an increasing number of countries have gradually come to accept it as an effective approach (Laloo and Hemalatha, 2011; Xiong et al., 2018). Shen-ling-bai-zhu-san (SL) is a classical spleen-tonifying Chinese herbal formula originating from “Tai Ping Hui Min He Ji Ju Fang”, which is composed of ten herbs, mainly Panax ginseng, Poria cocos, and Atractylodes macrocephala. According to theories of TCM, SL was used to treat chronic diarrhea by replenishing qi, invigorating the spleen, and resolving dampness. Our recent study (Ji et al., 2019) showed that SL can ameliorate lactose-induced diarrhea, but the mechanisms remain poorly understood.

Indeed, emerging data suggest that the gut microbiota is a vital “organ” for the absorption and metabolism of drugs, especially herbal medicine formulae. SL contain many tonifying herbs, such as ginseng, Poria cocos, Rhizoma Atractylodis and Chinese yam, which are largely composed of polysaccharides and considered to be prebiotics in modulating the gut microbiota. Increasing evidences have shown that herbals, including SL, that contain fiber, polysaccharide (PL), phenol, and other substances, have been reported to yield anti-obese, anti-diabetic and anti-inflammatory effects through the modulation of diverse gut microorganisms (Chang et al., 2015; Zhou et al., 2016; Lv et al., 2017; Zhang et al., 2018; Lv et al., 2019; Wu et al., 2019; Zhang et al., 2019; Feng et al., 2020; Yin et al., 2021). Therefore, we hypothesized that SL and PL derived from SL could alleviate lactose-induced diarrhea by modulating the community structure of the gut microbiota and, as a result, fermentation in the colon. In this study, we aimed to investigate changes in the composition of the luminal and mucosal microflora and the microbial fermentation capability of the gut microbiota by quantifying lactose-derived SCFAs in the cecal contents and portal blood after treatment. Finally, we utilized prediction software tools to estimate the potential interaction between intestinal bacteria and metabolic functions and investigated the correlation of changed luminal genera with lactose fermentation and changes in the mucosal genera with ion transporters.

## Materials and Methods

### Animals and Diarrhea Induction Using an Incremental High Lactose Diet

The experimental procedures followed the guidelines and practices of the Animal Care Ethics Committee of Xiyuan Hospital (Permission code: 2020XLC014). The procedures were conducted in accordance with the Beijing Administration Office Committee of Laboratory Animals. Male SD rats (200–220 g) were housed under an artificial 12 h light-12 dark cycle (lights on at 08:00 h). Forty rats were fed a standard chow diet *ad libitum* and had free access to water for 4 days prior to the induction of diarrhea.

### Preparation of SL and PL Derived From SL

SL, composed of ten Chinese medicinal herbs, was provided and quality controlled by Handian Pharmaceutical Co., Ltd. The volatile oil was extracted by frying Atractylodes macrocephala Koidz (400 g) with Amomum kernel (200 g). The residue of that extraction and eight others herbs, including Panax ginseng C. A. Mey (400 g), Poria cocos (Schw.) Wolf (400 g), *Dioscorea* opposita Thunb (400 g), Dolichos lablab L (300 g), *Nelumbo nucifera* Gaertn (200 g), Coix lacryma-jobi L. var. mayuen (200 g), Platycodon grandiflorum (Jacq.) A. DC (200 g), and *Glycyrrhiza* glabra L (400 g), were decocted in water three times. Then, the SL extract was filtered and concentrated to 1.20 g/ml. The mixture of ten herbs was refluxed with 10-fold distilled water at 100°C for 2 h. The extraction was repeated twice and the extracted solutions were concentrated and precipitated with 95% ethanol. The generated precipitate was then statically settled overnight and dried under vacuum, yielding the crude polysaccharide. The ratio of crude PL/SL was approximately 13% (Pang et al., 2007; Chen et al., 2019).

### Analysis of the Main Components of SL

The chemical components of SL were analyzed using an Agilent 1,200 high-performance liquid chromatography (HPLC) system (Agilent USA). SL components were separated by a CAPCELL PAK C18 MG II (150 mm × 4.6 mm, 5 μm). Three grams of SL powder was added to 30 ml of water-saturated n-butanol solution and incubated overnight. After 30 min of ultrasonic treatment, the filtrate was filtered again. After the filtrate was dried, 10 ml of 80% methanol was added to dissolve the residue, which was filtered with a 0.45 μm microporous filter membrane to yield the test solution. Ginsenoside Re (National Institutes for Food and Drug Control, NIFDC, 110754–200320), ginsenoside Rg1 (NIFDC, 110703–200322), ginsenoside Rb1 (NIFDC, 110704–200318), and atractylenolide Ⅰ (NIFDC, 11975–201501). The mobile phase consisted of acetonitrile (A) and 0.5% Trifluoroacetic Acid (TFA) water (B) for detecting ginsenoside Re, Rg1 and Rb1 at a flow rate of 1.0 ml min^−1^. The mobile phase was acetonitrile (A) and water (B) for detecting atractylenolide. A gradient program was used as follows: 18–22% A at 0–24 min, 22–29% A at 24–30 min, 29–33% A at 30–40 min, and 33–38% A at 40–50 min.

### Experimental Design

#### The Two Experimental Procedures (I and II) Were as Follows

**FIGURE 1:**
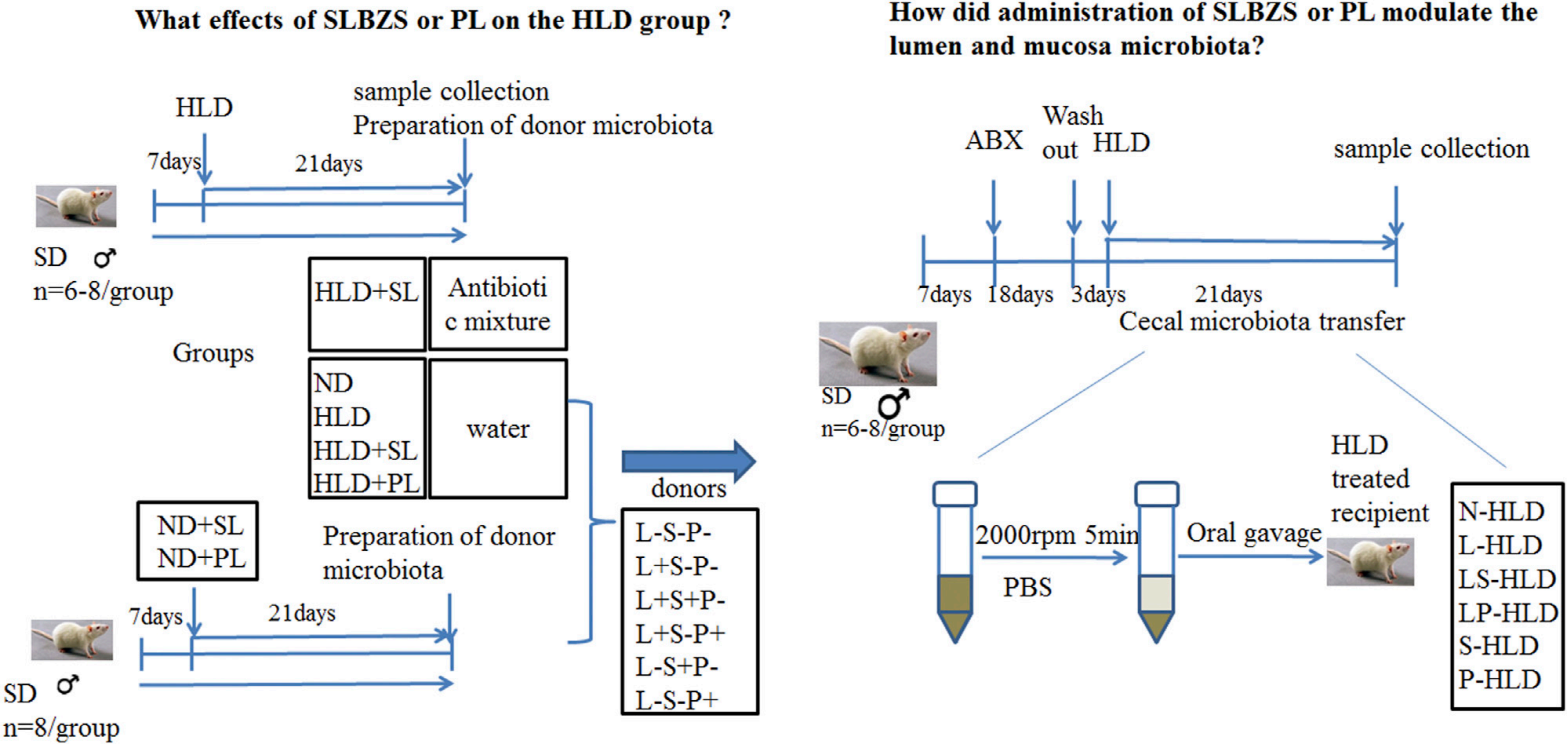
Schematic of the experimental design.

I To evaluate the effect of SL and PL**-**derived from SL on lactose induced diarrhea, 48 rats were randomly divided into seven groups (*n* = 6-8 for each group): a blank control group with normal chow diet (the saline group, ND), a model group with high lactose diet (HLD), an SL-treated group with high lactose diet (SL), an SL-treated group with normal diet (SN), a polysaccharide-treated diarrheal group with high lactose (PL), a polysaccharide-treated diarrheal group with normal diet (PN), and an SL-treated antibiotic group (STA). Chronic diarrhea was induced by supplementing the diet with an incremental high lactose diet (30%-40%–50%) lactose for 21 days, which consisted of 30% lactose for the first week, 40% lactose for the second week and 50% lactose for the third week, as described previously. The SL and PL groups were fed with incremental high lactose diet by oral gavage of SL (1 g/kg) and PL (0.13 g/kg) for 21 days once a day. The SN and PN group was fed with normal diet by intragastric administration of SL (1 g/kg) and PL (0.13 g/kg) for 21 days once a day, respectively. The STA group was fed with incremental high lactose diet and a cocktail of broad spectrum antibiotics (ABX) by oral gavage of SL (1 g/kg) for 21 days once a day. The detailed experimental procedure is shown in Figure 1. The rats were sacrificed by cervical dislocation under ether anesthesia on the 21st day.

### Antibiotic Administration and Cecal Microbiota Transplantation

II To evaluate the role of the gut microbiota in the anti-diarrheal effects of SL and PL, 46 rats were randomly divided into six groups (*n* = 6-8 for each group) according to different donor rats as follows: Lactose-SL-PL-, or L-S-P-; Lactose + SL-PL-, or L + S-P-; Lactose + SL + PL-, or L + S + P-; Lactose + SL-PL+, or L + S-P+; Lactose-SL + PL-, or L-S + P-; and Lactose- SL-PL+, or L-S-P+. The L + S-P-, L + S + P-, L + S-P+ groups were fed by high lactose diet to induce chronic diarrhea whereas the L-S + P-, L-S-P+ and L-S-P- groups received normal diet. Rats in the L + S + P- and L-S + P- groups were treated with SL (1 g/kg), rats in the L + S-P+ and L-S-P+ groups were administered with PL (0.13 g/kg), rats in the L-S-P- group was treated with 1 ml normal saline orally once per day.

All were randomized to receive donor cecal material via serial oral gavage (1,000 µl once daily for 21 days), and following transplantation, the cecal contents of each group were collected. A cocktail of antibiotics, including ampicillin (1 g/L), vancomycin (500 mg/L), ciprofloxacin HCl (20 mg/L), metronidazole (1 g/L), and neomycin (1 g/L) was added to the drinking water of rats in the antibiotic group and CMT groups for 18 days (Chen et al., 2019; O'Connor et al., 2019). After treatment, the fecal pellets were collected and the DNA content decreased to 10% of the control (data not shown), indicating that the antibiotic treatment was effective in removing most gut bacteria. The CMT groups received a washout period of autoclaved water for 3 days, and the antibiotic groups (n = 8) remained on a cocktail of broad spectrum antibiotics (ABX). After antibiotic treatment, 16S rRNA was measured in the cecal contents to ensure that the effects of antibiotics on the microbiota were similar.

### Preparation of the Microbiota of the Donor Group

The donor rats were from all six groups, including N + S-P-(N-HLD), L + S-P-(L-HLD), L + S + P-(LS-HLD), L + S-P+ (LP-HLD), L-S + P- (S-HLD) and L-S-P+ (P-HLD). The cecal and colon contents were collected, and 2 g samples were stored in a sterile cryotube for the detection of the microbiota. The remaining samples were diluted in pre-reduced phosphate buffered saline (PBS) (1:2 volume [contents]/volume [PBS]) and centrifuged at 2,000 rpm for 5 min under a laminar flow hood in sterile conditions, and the supernatant was collected and used as transplant material and quickly stored at -80°C until use in oral gavage (Allen et al., 2018; Chen et al., 2019; O'Connor et al., 2019).

### DNA Extraction and PCR Amplification

Microbial community genomic DNA was extracted from luminal and mucosal microbiota using the E. Z.N.A.® Soil DNA Kit (Omega Bio-Tek, Norcross, GA, U.S.) according to the manufacturer’s instructions. The DNA extract was checked on a 1% agarose gel, and DNA concentration and purity were determined with a NanoDrop 2000 UV–vis spectrophotometer (Thermo Scientific, Wilmington, USA). The hypervariable region V3-V4 of the bacterial 16S rRNA gene was amplified with the primer pairs 338F (5′-ACT​CCT​ACG​GGA​GGC​AGC​AG-3′) and 806R (5′-GGACTACHVGGGTWTCTAAT-3′) by an ABI GeneAmp® 9700 PCR thermocycler (ABI, CA, USA). PCR amplification of the 16S rRNA gene was performed as follows: initial denaturation at 95°C for 3 min, followed by 27 cycles of denaturing at 95°C for 30 s, annealing at 55°C for 30 s and extension at 72°C for 45 s, with a single extension at 72°C for 10 min, ending at 4°C. The PCR mixtures contained 4 μL of 5 × *TransStart* FastPfu buffer, 2.5 mM dNTPs 2 μl, 0.8 μl of forward primer (5 μM), 0.8 μl of reverse primer (5 μM), 0.4 μL of *TransStart* FastPfu DNA polysaccharide, 10 ng of template DNA, and ddH_2_O up to 20 μl. PCRs were performed in triplicate. The PCR product was extracted from a 2% agarose gel, purified using the AxyPrep DNA Gel Extraction Kit (Axygen Biosciences, Union City, CA, USA) according to the manufacturer’s instructions and quantified using a Quantus™ Fluorometer (Promega, USA).

### 16S rDNA Gene High-Throughput Sequencing

The V3-V4 region was amplified with the 16S rDNA gene universal primers 338 F (5′-ACT​CCT​ACG​GGA​GGC​AGC-3′) and 806R (5′-GGACTACHVGGGTWTCTAAT-3′). The PCR products were then purified, and the concentrations were adjusted. Sequencing was performed on an Illumina MiSeq PE300 system (MajorBio Co., Ltd., Shanghai, China). The raw data were deposited into the NCBI Sequence Read Archive (SRA). The accession number is SUB11116044.

### Data Analyses

The raw 16S rRNA gene sequencing reads were demultiplexed, quality-filtered by fastp version 0.20.0 (Chen et al., 2018)and merged by FLASH version 1.2.7 (Magoč and Salzberg, 2011) with the following criteria: 1) the 300 bp reads were truncated at any site receiving an average quality score of <20 over a 50 bp sliding window, the truncated reads shorter than 50 bp were discarded, and the reads containing ambiguous characters were also discarded; 2) only overlapping sequences longer than 10 bp were assembled according to their overlapped sequence. The maximum mismatch ratio of the overlap region is 0.2. Reads that could not be assembled were discarded. 3) Samples were distinguished according to the barcode and primers, and the sequence direction was adjusted, with exact barcode matching and two nucleotide mismatches in primer matching.

Operational taxonomic units (OTUs) with a 97% similarity cut off (Stackebrandt and Goebel, 1994; Edgar, 2013) were clustered using UPARSE version 7.1, and chimeric sequences were identified and removed. The taxonomy of each OTU representative sequence was analyzed by RDP Classifier version 2.2 (Wang et al., 2007) against the 16S rRNA database (e.g., Silva v138) using a confidence threshold of 0.7.

### Blood, Cecal Contents, Colonic Mucosa Sampling, and pH Measurement

Portal blood (2 ml) was sampled directly from the hepatic vein using a heparinized syringe. Blood samples were centrifuged for 4 min at 4°C at 600 x g. A volume of 500 µl of the plasma fraction was stored at -80°C. The colonic tissues were washed in ice-cold 0.9% (w/v) NaCl (pH 7.0), and the mucosa were scraped off using a glass slide before immediate freezing in liquid N2 and storage at -80°C until microbiota analysis and Western blotting. The cecal contents were sampled in sterile tubes. Samples were stored frozen at -80°C until analysis. Thawed feces (0.5 g) were mixed with 100 ml distilled water and stirred until dissolved. The samples were then soaked for 30 min at room temperature. The supernatant was used for pH examination by a pH meter (METTLER TOLEDO, S210-B).

### SCFA, Lactose, and Lactic Acid Quantity

The sample (∼50 mg) was removed from the freezer after being thawed at 4°C for 30 min, placed into a 1.5 ml centrifuge tube and accurately weighed. Then, 1 ml of 50% methanol aqueous solution was added to the tube and mixed for 30 min. The tube was then centrifuged at 4°C and 12,000 rpm for 5 min. The supernatant (50 μL), propionic acid isotope standard solution (50 μL, 5 μg/ml), 3-nitrophenylhydrazine (50 μL, 250 mM) methanol/water (1:1, v/v) solution and 1-(3-dimethylaminopropyl)-3-ethylcarbodiimide hydrochloride (50 μL, 150 mM)/methanol/water/pyridine (69.375:23.125:7.5) solution were charged into a 1.5 ml centrifuge tube and mixed at 30°C for 30 min. After that, butylated hydroxytoluene methanol solution (50 μL, 2 mg/ml) and methanol/water (250 μL, 3:1, v/v) solution were added to the tube and mixed. The tube was centrifuged at 4°C and 12,000 rpm for 5 min. The supernatant (200 μL) was transferred into a vial for MS detection. A Waters ACQUITY UPLC I-CLASS chromatography (Waters USA) unit equipped with a Waters UPLC BEH C8 column (2.1 mm (inner diameter) ×100 mm (length), 1.7 μm (particle dimension) was used for separation with a column temperature at 45°C. The mobile phase consisted of 0.01% formic acid aqueous solution (phase A) and methanol/isopropanol (8:2, v/v, phase B) with a flow rate of 0.3 ml/min, and the elution gradient used as follows: 95% A,5% B at 0–2 min; 85% A,15%B at 2–9 min; 0% A,100% B at 9–11 min; and 95% A, 5% B at 11–13 min. The injection volume was 5.0 µL. The MS data were collected by a Waters XEVO TQ-S Micro system. The parameters were set as follows: an ion source voltage of 3.0 kV, ion source temperature of 150°C, desolvation temperature of 350°C, desolvation gas flow of 1,000 L/h, and cone gas flow of 10 L/h.

Lactose and lactate were detected by corresponding assay kits (MAK017 and CAK1177, respectively (Sigma-Aldrich, St. Louis. MO, USA; Cohesion Biosciences, London, UK) according to the instructions. An aliquot of 50 mg of cecal and proximal colon content was dissolved in deionized water, vortexed for 20 s and centrifuged at 12,000 × *g* for 10 min at 4°C. The supernatant was eluted from HLB cartridges and evaporated to dryness under a nitrogen stream. Then, 50 ml of deionized water was added to the tube and redissolved.

### Western Blotting

Proximal colonic mucosa was collected from the colon mucosa of diverse groups. Tissues were homogenized and sonicated in cold lysis buffer with protein inhibitors and phosphatase inhibitors (Beyotime Biotechnology, Beijing). After 30 min of standing at 4°C, the samples were centrifuged at 12,000 x g for 30 min at 4°C. The pellets were discarded, and the supernatant was used for blotting. The proteins (30 µg) were separated via eight or 10% SDS/PAGE. The blot was washed with Tris-buffered saline containing Tween-20 and incubated overnight at 4°C with polyclonal primary antibodies against MCT1, sMCT1, slc26a3, NHE3, and pNHE3 (Supplementary Table S1) and the rabbit polyclonal anti-GAPDH antibody (Supplementary Table S1). After washing with TBST, the membranes were incubated with secondary antibodies (Supplementary Table S1).

### Statistical Analysis

All data are presented as the mean ± SEM. Differences among multiple groups were evaluated using ANOVA with Prism software (version 9.0.0, SAS Institute, Inc.), and those between two groups were assessed using the *t-test*.

## Results

### SL Chemical Component Analysis

Four chemicals were identified by HPLC as primary components of SL. The typical HPLC chromatogram of SL is shown in Supplementary Figure S1, and the ginsenoside Re, ginsenoside Rg1, ginsenoside Rb1, and atractylenolide Ⅰcontents in SL were 2.73 mg/g, 2.24 mg/g, 2.4 mg/g and 0.3 mg/g, respectively.

### SL, PL Treatment and CMT Alleviated Lactose-Induced Diarrhea

**FIGURE 2:**
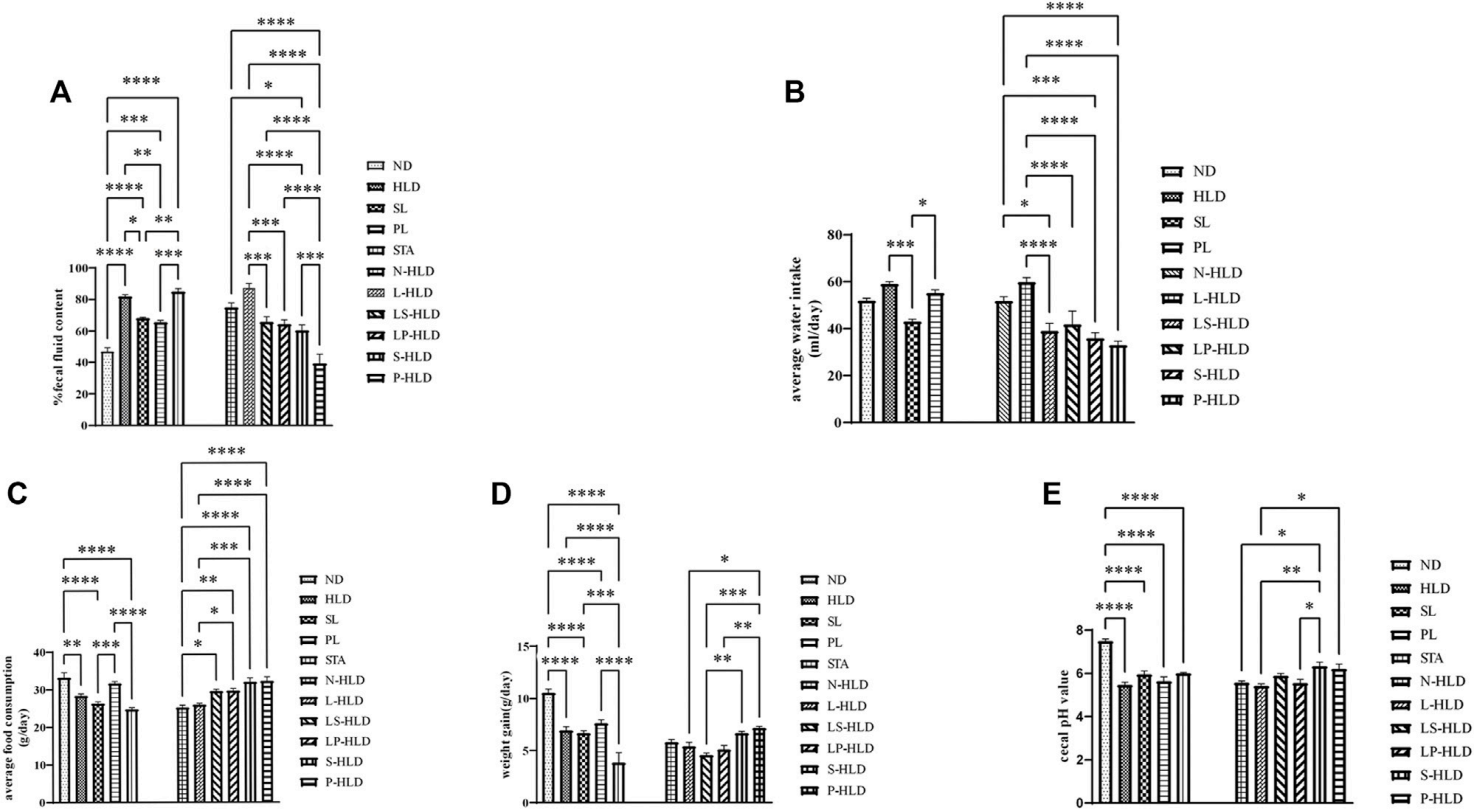
Effects of SL, polysaccharides derived from SL and CMT intervention on HLD rats **(A)** SL, polysaccharide and CMT intervention significantly reduced fecal fluid content **(B)** SL significantly reduced water intake compared to HLD group (left panel). LS-HLD, LP-HLD, S-HLD and P-HLD significantly decreased water intake compared to the L-HLD or N-HLD groups **(C)** Compared to the L-HLD group, S-HLD and P-HLD significantly increased average food consumption, promoted weight gain **(D)** and increased the cecal pH value **(E)**. Statistical significance was determined using one-way ANOVA, followed by Tukey’s test. *p ≤ 0.05, **p ≤ 0.01, and ***p ≤ 0.001 n = 6-8 per group.

A diarrhea rat model was induced by the administration of a 30%/40%/50% lactose diet for 21 days, according to our previous study (Xue et al., 2020). During the experimental process, high lactose resulted in weight loss and diarrhea compared with the rats without a lactose diet. The SL, S-HLD and P-HLD groups ameliorated the severity of lactose-induced diarrhea and decreased the concentration of fecal water, as shown in Figure 2A. In the CMT groups, LS-HLD, LP-HLD, S-HLD and P-HLD groups also significantly decreased the fecal water content compared to the N-HLD and L-HLD groups (Figure 2A). In contrast, SL did not improve the state of diarrhea after pretreatment with antibiotic but caused more severe watery stools, suggesting that gut microbiota play an important role in the process of SL treatment in diarrhea (Figure 2A). Furthermore, the model group showed a significant increase in water intake compared with the control group, which showed a remarkably decreased water intake after SL and PL treatments. Meanwhile, LS-HLD, LP-HLD, S-HLD and P-HLD showed lower water intake compared with the N-HLD and L-HLD groups (Figure 2B). The average daily food consumption was significantly increased compared with that of the HLD, N-HLD and L-HLD groups after PL, LS-HLD, LP-HLD, S-HLD and P-HLD treatments (Figure 2C). Remarkably, the S-HLD and P-HLD groups showed a reduction in body weight loss compared to the L-HLD group (Figure 2D). Chronic HLD treatment caused a reduction in body weight, which showed a slight increase following an SL treatment. The pH of fecal content in model rats was significantly decreased compared with that of normal rats, SL- and PL-treatment showed a slight pH increase, and S-HLD and P-HLD showed a significantly increase pH value compared to those of the N-HLD and L-HLD group (Figure 2E).

### Overall Structural Alteration Modulation of Luminal and Mucosa Microbiota After SL-, PL- and CMT Treatment

**FIGURE 3:**
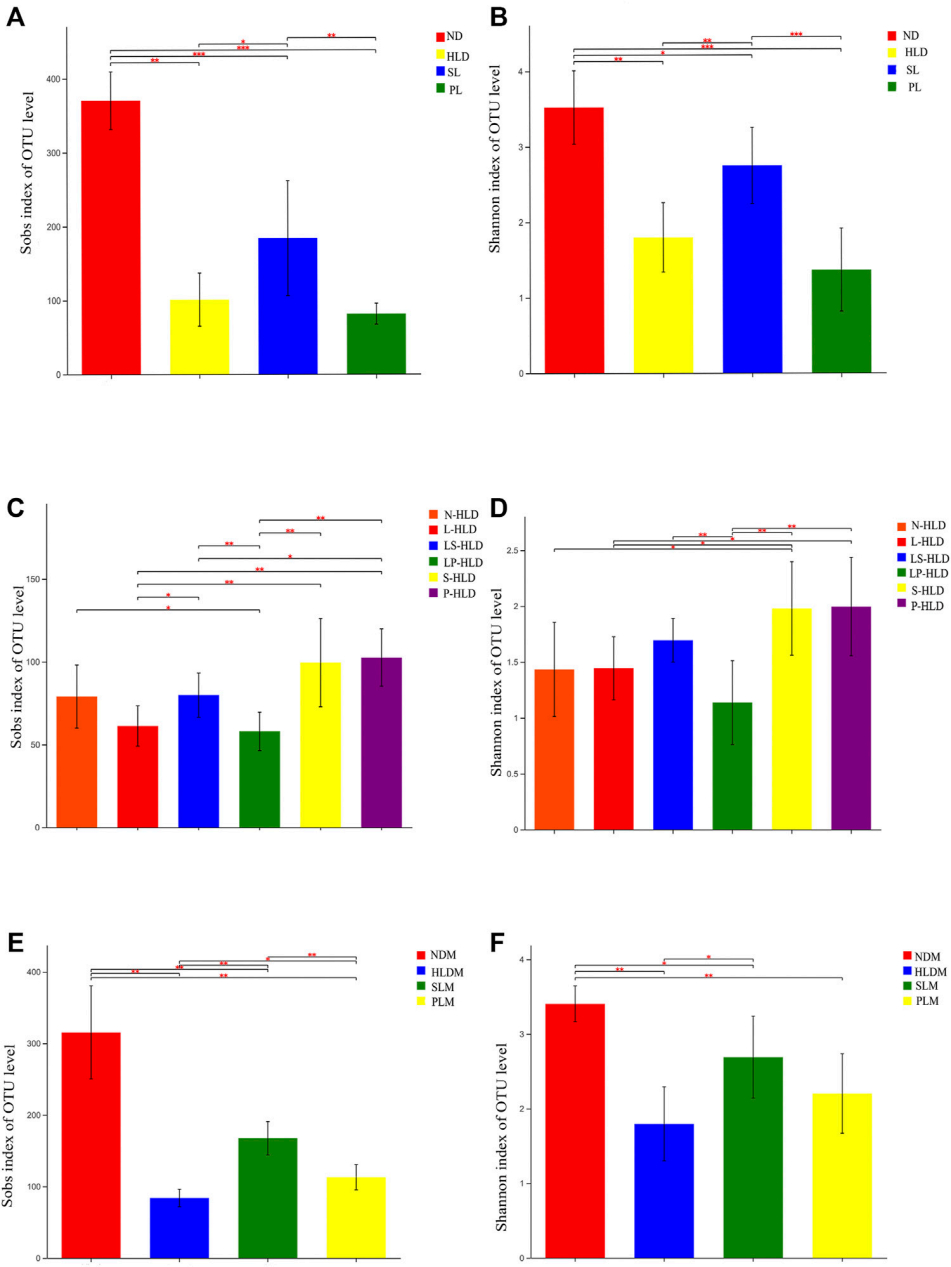
Alpha diversity of luminal and mucosal bacterial communities among distinct groups. The luminal **(A,C)** and mucosal **(E,F)** bacterial richness in the colon estimated by the Sob and Shannon indices after SL, PL and CMT. The luminal **(B,D)** and mucosal **(E,F)** bacterial diversity estimated by the Sob and Shannon indices among separate groups. Statistical significance was determined using the Wilcoxon rank-sum test. *p ≤ 0.05, **p ≤ 0.01, ***p ≤ 0.001, and ****p ≤ 0.0001, n = 6–8/group.

Since mucosa-associated bacteria live in closer proximity to the intestinal epithelium, they likely execute contrasting functions within the GI ecosystem compared with luminal microbiota. Therefore, the luminal and mucosal microbiota of rats were investigated by sequencing the bacterial 16S rRNA V3-V4 region. A total of 6-8 samples from each of the nine groups were analyzed by high-throughput pyrosequencing, which generated 5,286,598 useable reads, and 1,503 OTUs were collected. After treatment, the Sobs and Shannon indices of the luminal microbiota were higher in the SL and normal groups than in the HLD group (Figures 3A,B). Furthermore, the Sobs and Shannon indices of S-HLD were also higher than those of L-HLD (Figures 3C,D). The Shannon index of S-HLD was significantly increased than that of N-HLD (Figure 3D). There was no meaningful change in ecological diversity indices, such as Sobs and Shannon, after PL treatment compared to the HLD group (Figures 3A,B). In the P-HLD group, the Shannon and Sobs indices were significantly higher than those in the L-HLD group and slightly higher than those in the N-HLD group (Figures 3C,D), although the difference did not reach the significance. In the CMT group, the S-HLD and P-HLD groups showed a higher richness and diversity of gut microbiota than the L-HLD, LS-HLD and LP-HLD groups. After transplant with microbiota of untreated model rats, the Sobs index of gut microbiota was lower than that of the HLD model. Following SL-treated transplant, the Sobs and Shannon indices increased, suggesting that gut microbiota play an important role in the development of diarrhea and that SL alleviated diarrhea by modifying the community structure of the gut microbiota (Figures 3C,D). As shown in Figures 3E,F, the Shannon and Sobs indices of the mucosal microbiota in the HLD model were significantly lower than those in the normal group. The group of SL and PL significantly increased the Shannon and Sobs indices compared with the HLD model group, although it did not reach the normal level.

### Key Phylotypes of Luminal and Mucosal Microbiota Modulated by SL and PL

**FIGURE 4:**
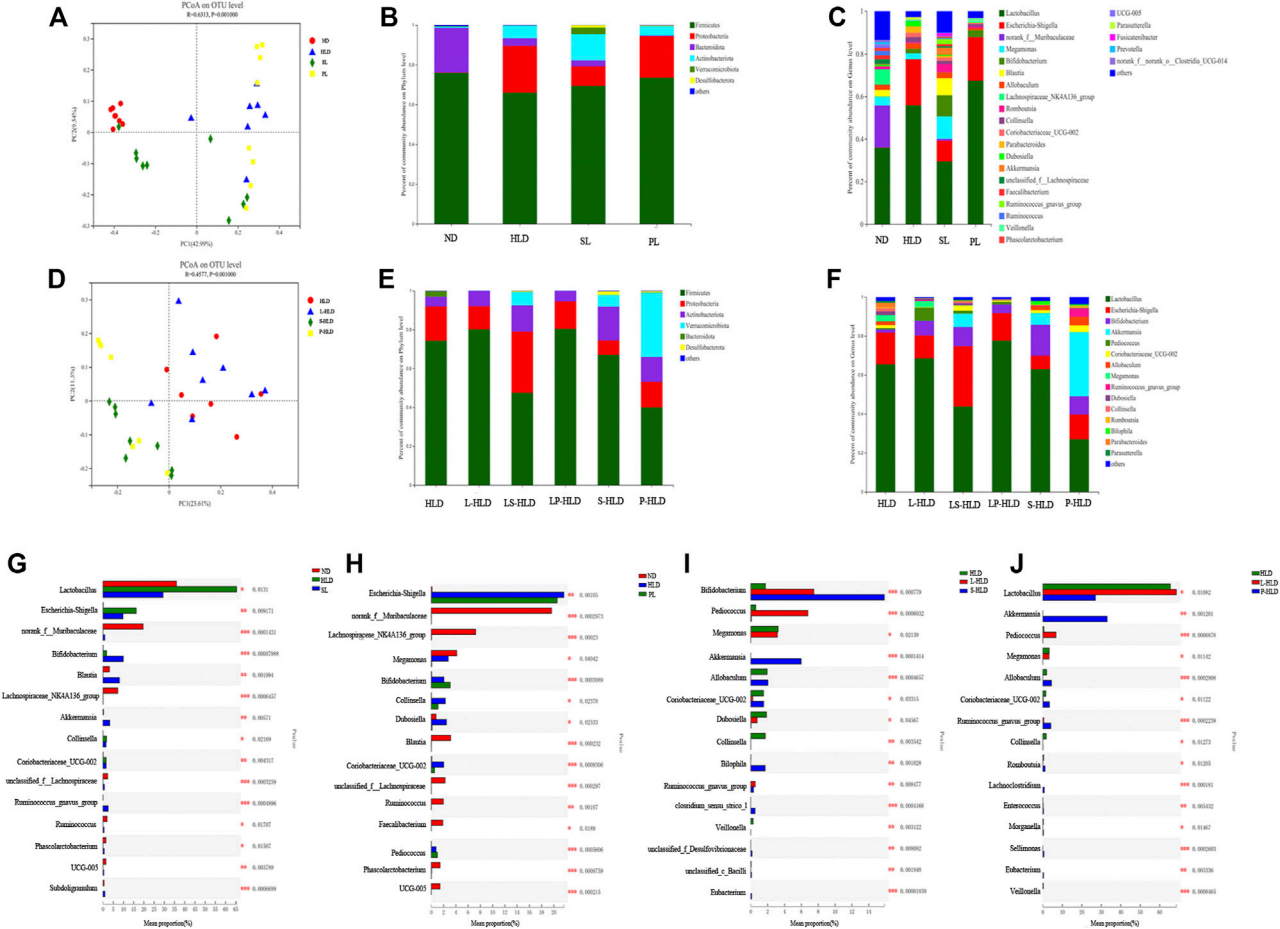
Effects of different treatments on luminal bacterial community structure. UniFrac-based PCoA of the colonic bacterial community at the OTU level under SL **(A)**, PL **(A)** and CMT treatment **(D)**. Relative abundance of luminal bacteria at the phylum level **(B,E)** and genus level **(C,F)** among distinct groups. Comparison of dominant genera under different treatments **(G–J)**.

Principal component analysis (PCoA) based on unweighted UniFrac showed a distinct clustering of bacterial types for each experimental group, suggesting that the SL, PL, and HLD groups caused major changes in gut microbiome profiles (R = 0.65, *p* = 0.001). The bacterial communities in the lumen microbiota and the HLD and SL groups had a higher degree of separation, suggesting that the overall structures of the bacterial communities in the groups were significantly different. In comparison, the SL and PL groups showed a partially overlapping microbiota, and the bacterial communities from two samples of the PL group overlapped with the HLD group (Figure 4A). Among the CMT group, PCoA revealed that the four groups formed two clusters in the ordination plot: one cluster belonged to the HLD and L-HLD groups, and the other belonged to the S-HLD and P-HLD groups (Figure 4D).

The compositions of colonic bacteria at the phylum and genus levels were measured and are shown in Figures 4B,C. Compared with the normal group, a decreased level of the phylum Bacteroidetes and an increased level of the phylum Proteobacteria were observed in the HLD model. In contrast, SL showed a slightly increased abundance of Verrucomicrobia and Desulfobacterota from 0 to 3.29% and from 0 to 1.21%, respectively, and a significantly increased Actinobacteria from 6.3% to 13% but suppressed numbers of the Proteobacteria (from 23% to 9.88%) (Figure 4B). Compared to the model group, 15 genera displayed significantly different abundances between the HLD group and the SL group, indicating that the protective effect of SL may be mediated by a subset of bacterial taxa (Figure 4G). The relative abundances of *Lactobacillus* and Escherichia-Shigella were increased by HLD, while SL reversed these HLD-induced changes to a considerable extent (Figure 4C). In contrast, HLD decreased the abundances of Blautia, Akkermansia, unclassified_f_Lachnospiraceae, Ruminococcus_gnavus_group, UCG005 and Subdoligranulum, all of which were restored to normal or higher levels by SL. In addition, the abundance of Bifidobacterium in the SL and PL groups was increased significantly compared with that in the HLD group and control group. In the PL group, the relative abundance of a few genera was restored by polysaccharides (Figure 4H), but after transplantation, the abundances of the phyla Verrucomicrobia and Actinobacteria were significantly increased from 0 to 33% and from 6.3% to 12%, respectively (Figure 4E). The relative abundance of beneficial genera was increased significantly compared to that in the HLD group, including Akkermansia (0 versus 33%), Bifidobacterium (2.0% versus 9.2%), Allobaculum (2.5% versus 4.46%) and Romboutsia (0.5% versus 1.14%), and the relative abundance of *Lactobacillus* was restored to normal levels (55% versus 27%) (Figure 4F, Figure 4J), and Escherichia-Shigella decreased from 21% to 12% in the PL-HLD group. In the SL group, the relative abundance of Escherichia-Shigella decreased significantly (from 21% to 6.9%) and was then restored to a large extent, and the relative abundance of beneficial genera was increased compared to that in the HLD group, including Bifidobacterium (1.74% versus 15.8%), Akkermansia (0 versus 5.95%), and Bilophila (0 versus 1.7%) (Figures 4F,I).

**FIGURE 5:**
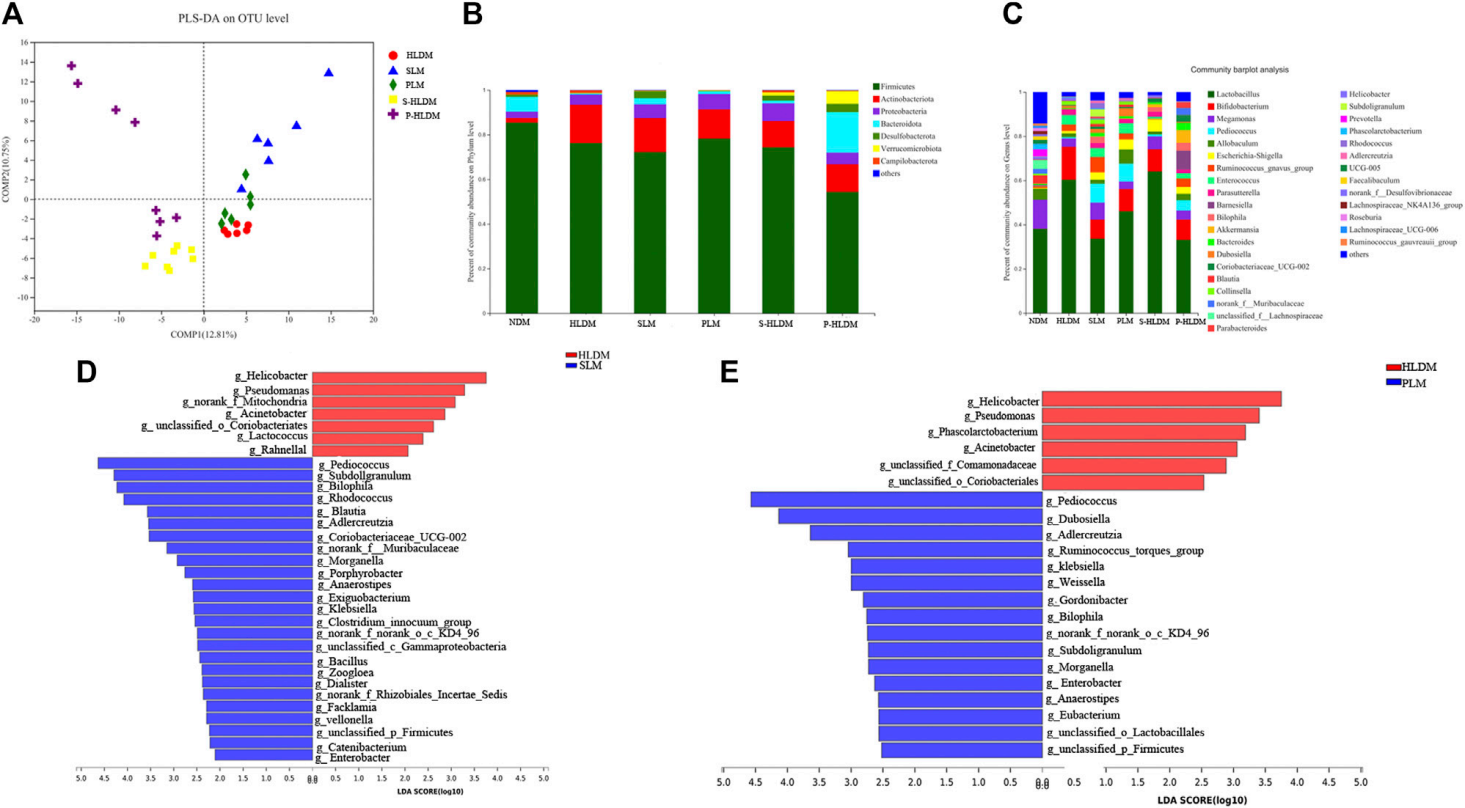
Effects of different treatments on mucosal bacterial community structure **(A)** PLS-DA score plots based on the relative abundances of microbial genera of different groups. Relative abundance of mucosal bacteria at the phylum level **(B)** and genus level **(C)** among distinct groups. Linear discriminant analysis effect size (LEfSe) analysis of the mucosal microbiota differences between HLD& SL **(D)** and HLD& PL **(E)** at the genus level.

Partial least squares discriminant analysis (PLS-DA), which is a supervised analysis suitable for high-dimensional data, was performed (Figure 5A). The mucosa microbiota results showed that HLD, SL, P-HLD and S-HLD had better separation and that HLD and PL had a slight degree of aggregation. Among the mucosal microbiota, the relative abundance of the Bacteroidota phylum was increased after SL, PL and CMT treatment (Figure 5B). The relative abundance of *Lactobacillus* in the SL, PL, and P-HLD groups was lower than that in the HLD group by 45%, 23% and 45%, respectively, but was subsequently restored to normal levels to a substantial extent (Figure 5C). Meanwhile, we also found a decreasing trend in the relative abundance of Bifidobacterium after treatment (15% HLD versus 8.6% SL, 10% PL, 10% S-HLD and 9.2% P-HLD) and a slightly increasing trend in the relative abundance of Escherichia-Shigella after SL, PL and CMT treatments (1.1% versus 3.3, 4.6, 5.5, 3.1%) (Figure 5C). In addition, a significant increase in Akkermansia from 0.11% to 1.4% and 5.8%, respectively, was detected in the S-HLD and P-HLD groups. The proportion of pediococcus tended to increase after SL, polysaccharide and CMT treatment, differing in degree (Figure 5C).

We compared the mucosa microbiota using Linear discriminant analysis Effect Size (LEfSe) to identify the specific bacterial taxa associated with the SL and PL. As shown in Figure 5D, the results revealed that Pediococcus, Subdoligranulum, Bilophila, Rhodococcus, Adlercreutzia, Zoogloea, Ralstonia, *Bacillus*, Facklamia, g__norank_f__Muribaculaceae, g__norank_f__Rhizobiales_Incertae_Sedis, Exiguobacterium, *Morganella*, Veillonella, and Anaerostipes were enriched in SL rats. In the PL group, the genera Pediococcus, Dubosiella, Adlercreutzia, Weissella, Ruminococcus_ torques_group, *Klebsiella*, Bilophila, *Enterobacter*, unclassified_p__Firmicutes, and unclassified_o__Lactobacillales were enriched (Figure 5E). In the S-HLD group, the genera Escherichia-Shigella, Burkholderia-Caballeronia-Paraburkholderia, and unclassified_c__Gammaproteobacteria were enriched (Supplementary Figure S2 left). The abundance of the following were increased in P-HLD group: Ruminococcus navus_group, g__Bilophila, g__norank_f__Muribaculaceae, g__Coriobacteriaceae_UCG-002, g__unclassified_f__Peptostreptococcaceae, g__Sellimonas, g__unclassified_c__Bacilli, g__Eubacterium,g__*Morganella*, g__unclassified_o__Lactobacillales,g__unclassified_p__Firmicutes, g__unclassified_f__Desulfovibrionaceae (Supplementary Figure S2 right).

### SL-, PL- and CMT Significantly Decreased the Concentrations of Lactose and Lactate Acid in the Cecal Luminal Contents

**FIGURE 6:**
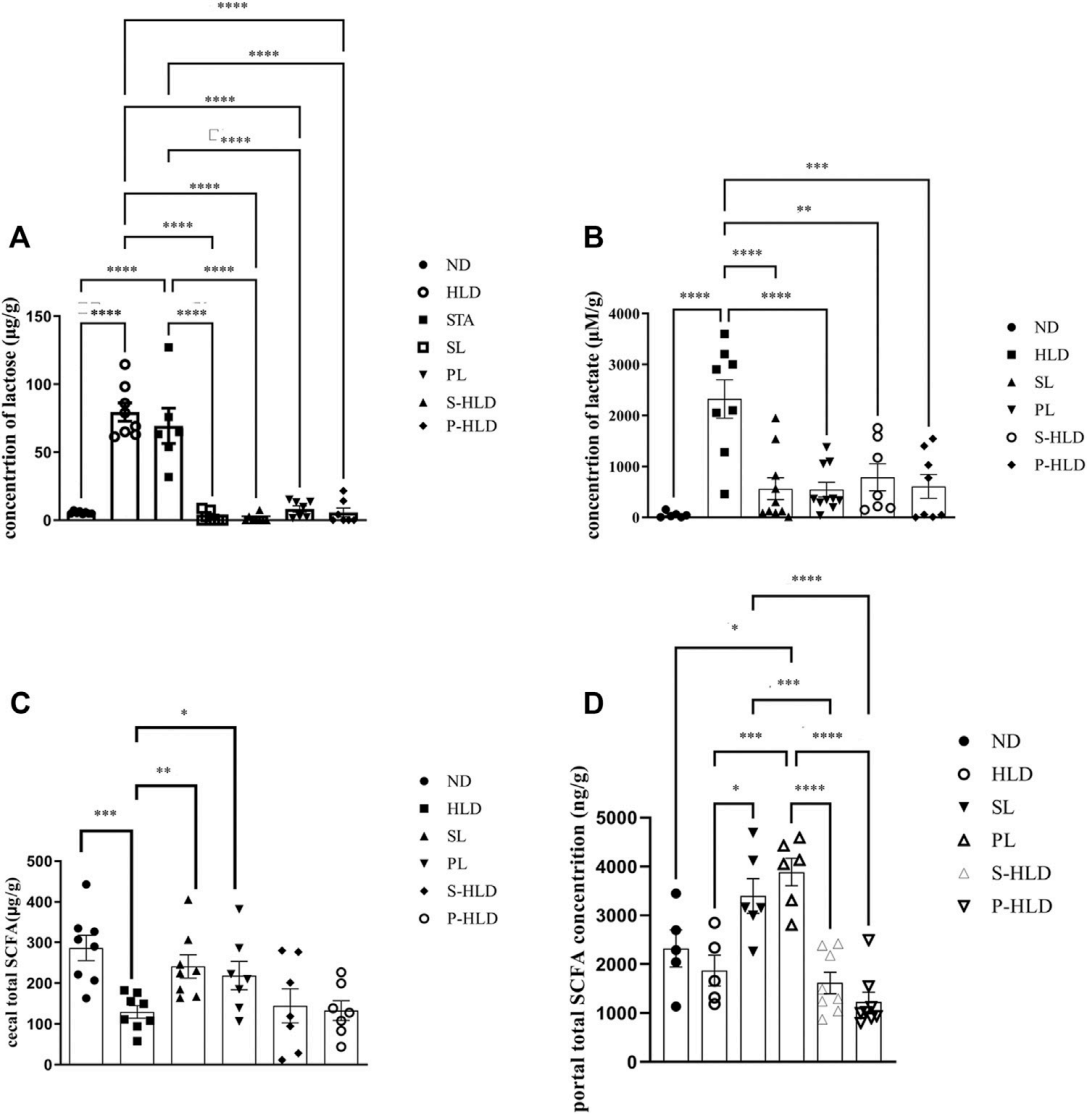
Concentration of cecal lactose **(A)**, lactic acid **(B)**, SCFA **(C)** and portal SCFA **(D)** under different treatments. Statistical significance was determined using a 2-tailed unpaired Student’s t test for two groups or one-way ANOVA for many groups, followed by Tukey’s test. *p ≤ 0.05, **p ≤ 0.01, ***p ≤ 0.001, and ****p ≤ 0.0001, n = 6-8 per group.

Due to natural lactase deficiency in adult rats, a large amount of undigested lactose accumulates in cecal luminal contents, which would elevate the osmotic load. The colonic microbiota, which ferment lactose and lactic acid, play a vital role in colonic processing of lactose. Higher amounts of lactose and lactic acid were mainly encountered in the cecum of the model group (Figures 6A,B). After SL-, PL- and CMT treatments, the concentration of cecal lactose was significantly decreased to normal levels, suggesting that these treatments reduced the fecal water content by accelerating the colonic fermentation of lactose. Although the concentration of lactic acid **could not be reversed to normal levels, it was significantly lower than that in the model group.** In the STA group, the concentration of lactose did not decrease, suggesting that the gut microbiota would be influenced by the treatment of SL.

### Changes in the Level of SCFAs in Cecal Luminal Content and Portal Vein Blood

SCFAs were quantified in intestinal contents and portal vein blood among distinct groups, including ND, HLD, PL, SL, S-HLD, and P-HLD treatments. The total SCFA in intestinal contents was 54% lower in the model group than in the normal group (Figure 6C), which was consistent with our previous study (Xue et al., 2020). In portal vein blood, there was no significant difference in the total SCFA concentration between the control and model groups. After SL or PL treatment, total SCFAs in intestinal contents were significantly increased by 81% and 66%, respectively, including acetate, propionate and lactate concentrations (Figure 6C, Supplementary Figure S3A–C). Total SCFAs in portal vein blood were also significantly increased by 90% and 146%, respectively, especially butyrate, as shown in Figure 6D and Supplementary Figure S3D–F.

No significant difference in SCFAs was noticed in intestinal contents between the CMT and model groups. A slight increase in SCFA (acetate) was observed in the S-HLD group, and butyrate was significantly increased in the P-HLD group compared to the HLD group (Figure 6C, Supplementary Figure S2). Finally, quantifications in the portal blood revealed a slight decrease in total SCFA in the S-HLD and P-HLD groups, mainly in butyrate concentration (Figure 6D Supplementary Figure S2).

### MCT1, sMCT1, NHE3, pNHE3, and slc26a3 Protein Expression

**FIGURE 7:**
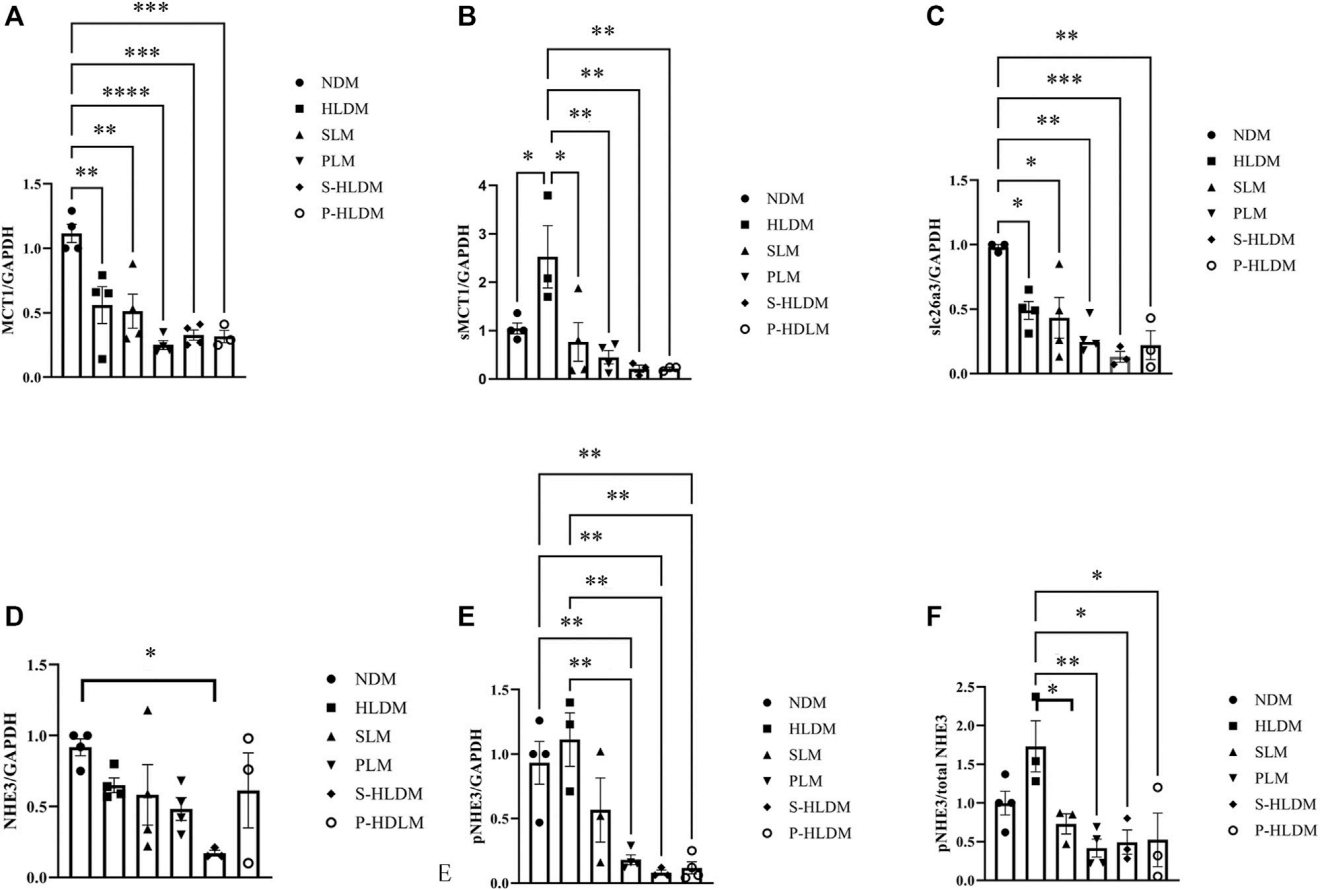
The expression levels of ion transporters were analyzed by Western blot, and the density of MCT **(A)**, sMCT1 **(B)**, SLC26A3 **(C)**, NHE3 **(D)**, pNHE3 **(E)** and pNHE3/NHE3 **(F)** was calculated as shown in different groups. Data are represented as the mean ± SEM (n = 3). *p ≤ 0.05, **p ≤ 0.01, ***p ≤ 0.001.

**FIGURE 8:**
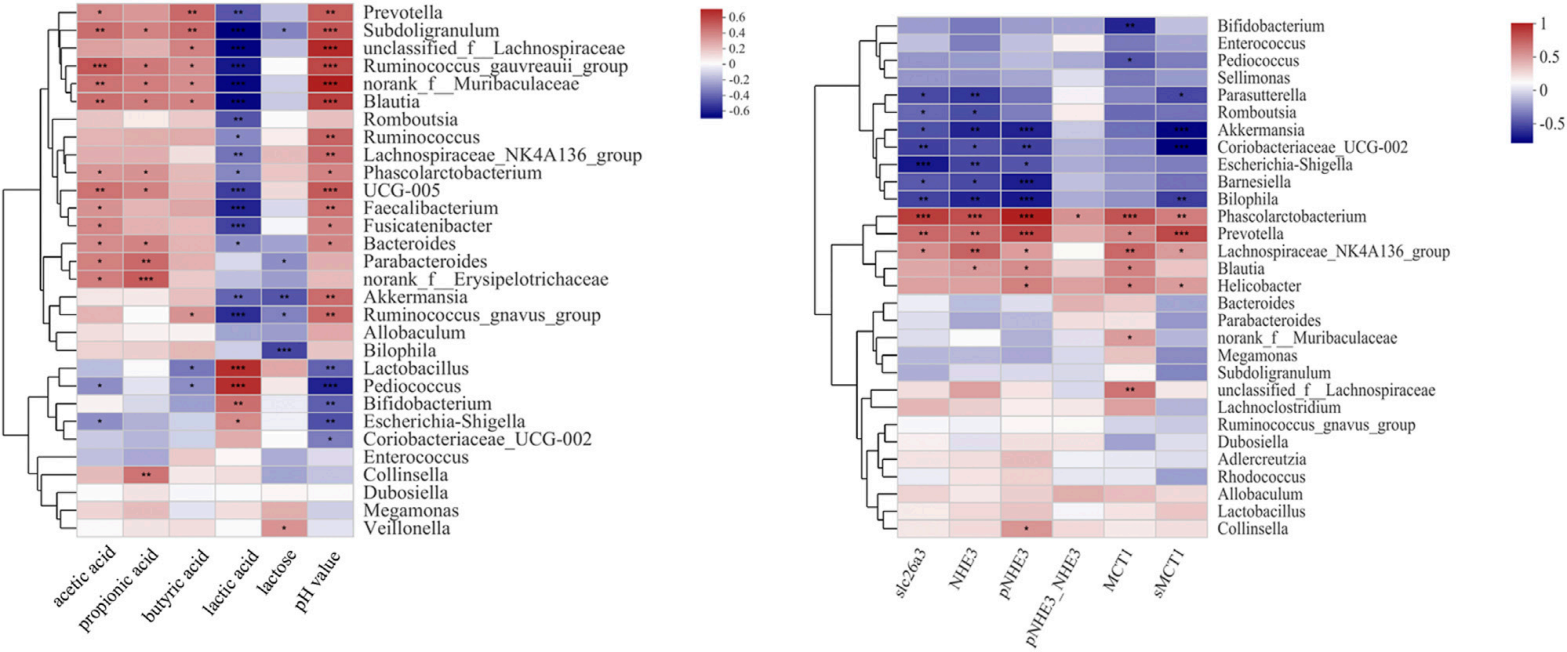
Correlation analysis between the 30 most dominant luminal genera in all samples and microenvironmental factors (left). Correlation analysis between the 30 most dominant mucosal genera in all samples and ion transporter expression (right) Data are presented as the mean ± SEM (n = 6). Cells marked with an asterisk depict significance following Spearman correlation and false discovery rate (FDR) correlation for multiple comparisons, *p ≤ 0.05, **p ≤ 0.01, ***p ≤ 0.001.

MCT1 and sMCT1 are the most likely major contributors to SCFA entry into the colonic epithelium. Both NHE3 and slca6a3 are prominently expressed in colonic epithelial cells and participate in the tandem operation of apical Na/H exchange and Cl/HCO3 exchange processes and electroneutral NaCl absorption. Figure 8 illustrates the effect of SL, PL and CMT on NHE3 pNHE3, MCT, sMCT1 and slc26a3 protein expression in the proximal colonic mucosa. The treatments did not significantly change MCT1 expression (Figure 7A). Compared to the control group, sMCT1 protein expression increased, but not significantly in the HLD group (Figure 7B). SL, PL and CMT significantly downregulated sMCT1 protein expression levels. slc26a3 protein expression decreased in the model group compared with that in the control group, but none of the treatments restored its expression (Figure 7C). The ratio of pNHE3/total NHE3 was high in the model group but was decreased after SL, PL and CMT treatments (Figure 7 D, E, F).

### The Correlations Between Luminal Microbes and Lactose Metabolism

The therapeutic effect of SL and PL on luminal microbial communities may primarily be mediated by the metabolites of lactose, such as lactate and SCFAs. The change in the protein expression of SCFA transporters and NHE3 may play a key role in the therapeutic effect of SL and PL on mucosa microbiota. To confirm the relationship between the altered gut luminal microbiota composition and lactose metabolism and between the altered mucosa microbiota composition and relevant transporter expression, we conducted Spearman’s correlation analysis displayed by a heatmap, as shown in Figures 8A,B. Figure 8A shows two distinct clusters based on the strong correlation between genera and metabolic parameters. Cluster 1, including Prevotella, Subdoligranulum, unclassified_f__Lachnospiraceae, Ruminococcus_gauvreauii_group, norank_f__Muribaculaceae, Blautia, Romboutsia, Ruminococcus, Lachnospiraceae_NK4A136_group, Phascolarctobacterium, UCG-005, Faecalibacterium, Fusicatenibacter, *Bacteroides*, Parabacteroides, norank_f__Erysipelotrichaceae, Akkermansia, Ruminococcus_gnavus_group, Allobaculum and Bilophila, showed a negative correlation with lactose and/or lactate and/or a positive correlation with SCFA and pH value. Cluster 2, including *Lactobacillus*, Bifidobacterium, *Lactobacillus*, Pediococcus, Coriobacteriaceae_UCG-002, Escherichia-Shigella, *Enterococcus*, Collinsella, Dubosiella, Megamonas and Veillonella, was negatively correlated with the SCFA concentration and pH value but positively correlated with the lactose and lactate concentrations.

### The Correlations Between Mucosal Microbes and Ion Transporter Expression

As shown in Figure 8B and Supplementary Figure S3, among the mucosa bacteria at the genus level, Phascolarctobacterium, Prevotella and Lachnospiraceae_NK4A136_group were positively correlated with the expression levels of slc26a3, NHE3, MCT1 and sMCT1. In contrast, Barnesiella, Parasutterella, Bilophila, Akkermansia, and Coriobacteriaceae_UCG-002 showed negative correlation with slc26a3, NHE3 and sMCT1. In addition, Phascolarctobacterium was positively correlated with the ratio of pNHE3/NHE3.

### SL, PL and CMT Treatments Influenced Metabolic Pathways of Gut Microbiota

o investigate the influence of SL, PL and CMT treatment on potential metabolic pathways of luminal and mucosal microbiota in HLD rats, we conducted phylogenetic investigation of communities by reconstruction of unobserved states (PICRUSt) analysis based on the luminal and mucosal microbiota composition of each group.

**FIGURE 9:**
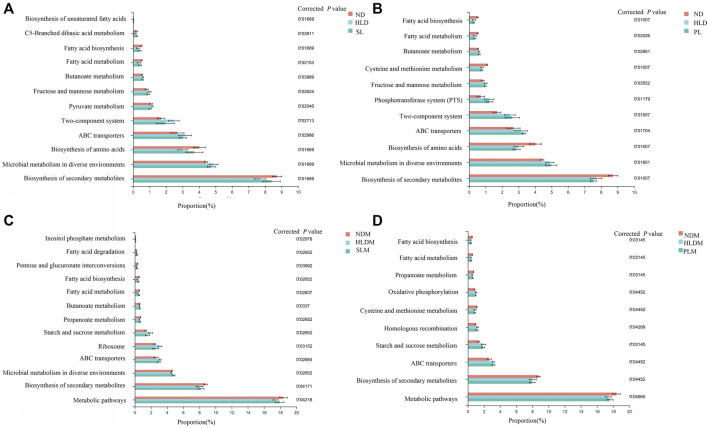
Functional prediction of altered luminal and mucosal microbiota based on KEGG pathways **(A)** A total of twelve significantly recovered KEGG pathways by SL treatment in the luminal microbiota of HLD rats **(B)** Few recovered KEGG pathways by polysaccharide treatment in luminal microbiota **(C)** A total of thirtheen significantly recovered KEGG pathways by SL treatment in mucosal microbiota of HLD rats **(D)** A total of eight significantly recovered KEGG pathways by PL treatment in the mucosal microbiota of HLD rats. The results were determined by a nonparametric factorial Kruskal–Wallis (KW) sum-rank test. Data are represented as the mean ± SEM (n = 6-8 per group).

As indicated in Figure 9A, among 268 Kyoto Encyclopedia of Genes and Genomes (KEGG) pathways at level 3 KEGG Orthologys, we found 12 pathways that were significantly different among the ND, HLD and SL groups, most of which were related to fatty acid metabolism and biosynthesis, pynruvate metabolism, Fructose and mannose metabolism, and biosynthesis of amino acids and secondary metabolites.

In the HLD group, fatty acid metabolism and biosynthesis, biosynthesis of amino acids and secondary metabolites were all significantly decreased; carbohydrate metabolism (Fructose and mannose metabolism, Butanoate metabolism, Pyruvate metabolism), Two-component system, ABC transporters and Microbial metabolism in diverse environments were significantly increased. SL restored 12 pathways, including fatty acid and carbohydrate metabolism and biosynthesis of fatty acid, amino acids and secondary metabolites, as shown in Figure 9A. In the mucosa sample, after SL treatment, the pathways of fatty acid metabolism and biosynthesis, carbohydrate metabolism (Propanoate metabolism, Starch and sucrose metabolism), Ribosome, ABC transporters and metabolic pathways were restored, as shown in Figure 9 C. Although PL treatment reversed a few metabolic pathways to normal levels in the luminal microbiota (Figure 9B), in the mucosal microbiota, some pathways, including fatty acid metabolism and biosynthesis, carbohydrate metabolism (Propanoate metabolism, Starch and sucrose metabolism) were restored to some extent (Figure 9D).

S-HLD resulted in upregulation of glycosphingolipid biosynthesis, N- glycan biosynthesis and other glycan degradation in the luminal microbiota (Supplementary Figure S4A). In the mucosal microbiota, S-HLD restored some pathways including fatty acid metabolism and biosynthesis, carbohydrate metabolism, translation and ABC transporter to some extent (Supplementary Figure S4C).P-HLD decreased the pathways of carbohydrate metabolism and quorum sensing, increased the pathways of biosynthesis of amino acids and secondary metabolites in the luminal microbiota, comparing to the L-HLD group (Supplementary Figure S4B). In the mucosal microbiota, the pathway of fatty acid metabolism and biosynthesis, carbohydrate metabolism and quorum sensing, ABC transporter, biosynthesis of amino acids and metabolic pathways were stored after PL transplantation (Supplementary Figure S4D).

## Discussion

In the present study, the effect of SL and SL-derived polysaccharide on high lactose-induced diarrhea was studied *in vivo.* The results demonstrated that SL improves the symptoms of diarrhea, as evidenced by a reduction in watery stool, fecal water content via the regulation of gut luminal microbiota fermentation and mucosa-associated bacterial populations, which may dramatically change in a setting of altered host ion transporters.

Our previous study indicated that SL ameliorated HLD-induced diarrhea associated with ion transport (Ji et al., 2019). It has also been reported that SL could regulate the gut microbiota in a functional dyspepsia model (Zhang et al., 2019). However, no relevant studies have reported that SL and PL derived from SL could ameliorate HLD-induced diarrhea by regulating gut microbiota. In the present study, we demonstrated the significance of the gut microbiota by analyzing alterations of gut luminal and mucosal microbiota in the cecum, colonic fermentation metabolites, and SCFA transporter and Na^+^ transporter expression and phosphorylation levels with and without SL and polysaccharide treatment.

We also sought to assess the effects of manipulation of the gut microbiota by chronic antibiotic administration on an HLD model with SL treatment and to investigate whether cecal microbiota of SL or PL treatment transfer could ameliorate the effects in ABX rats induced by HLD. Our results showed that after antibiotic administration, the effect of SL on HLD was not observed. The body weight decrease, water stool and fecal water increase in SL-treated ABX-rats, as compared to those of the HLD model without SL, suggests that the gut microbiota play a key role in the therapeutic effect of SL on the HLD model. Our study found that recipient rats receiving fecal transplantation from SL- and PL-treated HLD rats also showed a significant remission of HLD-induced diarrhea, which supports the beneficial effects of SL and PL that can be partially mediated by gut microbiota.

The effects of SL, PL and CMT on the composition of the luminal and mucosal microbiota of mice were investigated via high-throughput MiSeq sequencing. As reflected by the Sobs and Shannon indices, we observed that HLD reduces the alpha diversity of the gut microbial community. Although SL treatment could not recover the richness of gut microbiota to normal, the alpha diversity was significantly improved after SL treatment compared to that of the HLD model, suggesting that SL reverses the diarrhea-induced reduction in the species richness of gut microbiota. Previous studies demonstrated that SL could improve functional dyspepsia (Zhang et al., 2019), antibiotic-associated diarrhea (Lv et al., 2017), and inflammatory bowel disease (Lv et al., 2019) by regulating microbiome structural changes such as abundance of *Bacteroides* spp., Corynebacteriaceae, Lactobacillaceae, Paraprevotellaceae, Veillonellaceae, Prevotellaceae, Clostridiaceae, and others. In our study, after SL treatment, the abundances of the phyla *Firmicutes* and *Proteobacteria* were remarkably decreased. At the genus level, SL obviously increased the abundance of Bifidobacterium*,* Blautia, Megamonas and Akkermansia and greatly decreased the relative abundance of *Lactobacillus* and *Escherichia/Shigella.* It has been demonstrated that Bifidobacterium (adolescentis, animalis, bifidum, breve, and longum) and *Lactobacillus* (acidophilus, casei, fermentum, gasseri, johnsonii, paracasei, plantarum, rhamnosus, and salivarius) have become the most widely used species as probiotics. *Lactobacillus* spp. is evidently the most prominent probiotic agents among lactic acid bacteria (Turroni et al., 2014). It is generally believed that dietary lactose has a prebiotic effect on the gastrointestinal microbiota. Tran et al. showed that a high concentration of dietary lactose (20%) increased the relative abundance of Lactobacilli and reduced *E. coli* in the large intestine of piglets (Tran et al., 2012). Furthermore, lactose feeding increases the proportions of fecal Lactobacilli and Bifidobacteria in lactose maldigesters (Szilagyi et al., 2010; Ito and Kimura, 1993), which are considered to be colonic microbes adapting to the presence of lactose in the colonic lumen (Forsgård, 2019; Zhao et al., 2021). In our study, we found the similar results that included the relative abundance of *Lactobacillus* and Bifidobacterium being significantly increased in the HLD group compared to the control group.

We considered that the relative abundance of Lactobacilli was lower in the luminal microbiota after SL treatment because the concentration of luminal lactose was significantly reduced due to being hydrolyzed into lactate and SCFA, resulting in a decrease in lactose-fermenting bacteria, which is the consequence of the effect of SL on the gut microbiota. This led us to question how lactose is hydrolyzed by the microbiota. A possible explanation could be that SL induced a high proportion of Bifidobacteria, Blautia, Akkermansia and Ruminococcus gnavus, as carbohydrate bacteria (Pokusaeva et al., 2011; Forsgård, 2019; Liu et al., 2021). Like SL, S-HLD enhanced luminal bifidobacteria and Akkermansia and suppressed *Escherichia*/*Shigella*. In addition, the mucosal genera Pediococcus, Subdoligranulum, Bilophila, Rhodococcus, Blautia, Adlercreutzia, Coriobacteriaceae_UCG-002, and norank_f__Muribaculaceae were enriched in S-HLD compared to HLD group. Although the relative abundance of *Lactobacillus* in S-HLD group is not significantly different from the HLD group, the relative abundance of Bifidobacterium decrease from 15% to 10%, which also might be the consequence of the effect of S-HLD treatment on the gut microbiota. Further, according to our observation, the effect of SL on high-lactose induced diarrhea is better than that of S-HLD indicating the relative lower abundance of *Lactobacillus* in SL group compared to S-HLD group. Although the PL treatment cannot reverse the richness of gut luminal microbiota and few genera were restored, the richness of gut mucosa microbiota was higher and upregulated mucosal microorganisms such as g_Pediococcus, g_dubosella, Adlercreutzia, Ruminococcus_torques_group, Allobaculum, Weissella, Subdoligranulum, which are considered as the lactic acid bacteria or SCFA-producing bacteria. After transplantation, it is interesting that PL-HLD significantly reduced the relative abundance of *Lactobacillus* and increased the relative abundance of Akkermansia, Bifidobacterium, Allobaculum, and Ruminococcus gnavus compared to that of the HLD group.

Bifidobacteria can utilize a diverse range of dietary carbohydrates that escape degradation in the upper parts of the intestine and are believed to play a key role in carbohydrate fermentation in the colon (Pokusaeva et al., 2011). A recent study (Brandao Gois et al., 2022) found that Bifidobacterium abundance is significantly associated with the total gastrointestinal complaints score in lactose intolerant (LI) individuals. In our study, HLD increased the abundance of Bifidobacteria, and its proportion was further increased after SL, PL and CMT. We speculated that SL and PL enhanced different bifidobacterial strains with strong carbohydrate metabolic abilities; however, this will require additional study.

Furthermore, the relative abundance of Akkermansia, as a potentially beneficial bacterium, was significantly increased after SL and CMT treatment. Many previous reports have shown that traditional Chinese medicine, including rhein (Wang et al., 2018), Rhizoma Coptidis (Tan et al., 2016) and berberine (Zhu et al., 2018), which play a prebiotic role, could promote the function of Akkermansia, promote intestinal mucosal barrier repair, positively influence bacterial metabolism, and affect the “gut-liver axis” and “brain-gut axis”.

In our study, the concentration of cecal lactate was significantly decreased after SL and PL treatment compared to that in the HLD model, and it is interesting that the concentrations of cecal acetate and propionate were significantly increased and that their concentrations in serum were decreased. In contrast, the concentrations of cecal and serum butyrate were significantly increased. Although the relative abundance of lactic acid bacteria is high, meanwhile SL and PL also induced a high proportion of SCFA-producing bacteria such as Bifidobacteria, Megamonas, Blautia, Romboutsia, Allobaculum, Akkermansia which could promote lactic acid further converting to SCFA. So the lactic acid concentration is not high but SCFA is high in cecal content. Meanwhile, previous study *in vitro* (Jiang and Savaiano, 1997) has shown that lactic acid bacteria could shift bacterial metabolism from lactate to SCFA formation. So the lactic acid concentration is not high but SCFA is high in cecal content. High SCFA concentrations in the cecum may be explained either by active production and/or by low uptake. The total SCFA in cecal and portal blood was higher in the SL and PL treatment groups, suggesting that SL and PL participate in bacterial metabolism to SCFA as substrates, which play a prebiotic role.

The altered gut microbial composition is closely related to lactose fermentation, including lactate and SCFA production. SL increases luminal Bifidobacteria, Megamonas, Blautia, Romboutsia, Akkermansia and mucosal Pediococcus, Rhodococcus, and Coriobacteriaceae UCG-002, which are involved in carbohydrate metabolism and SCFA production. In agreement with the results from the literature (Guo et al., 2021; Huang et al., 2021; Yu et al., 2021) and our 16S rRNA pyrosequencing analysis, Spearman’s correlation assay further proved that the luminal genera Subdoligranulum, Blautia, Ruminococcus_torques_group and Norank_f_Muribaculaceae were positively correlated with cecal luminal SCFA concentrations, including acetate, protratate, butyrate and pH values, while they were negatively correlated with luminal lactate. It is worth mentioning that the genera Bilophila, Desulfovibrio, Alistipes, Akkermansia, and *Bacteroides* showed a strong negative correlation with luminal lactose, indicating that these genera might play an important role in lactose fermentation.

NHE3 activity has been shown to be regulated by dietary and humoral factors in a variety of Na^+^ transporting epithelia. It has been demonstrated that luminal SCFAs stimulate NHE3 activity (Gonda et al., 1999; Krishnan et al., 2003). In the kidney and gallbladder, higher NHE3 activity was associated with confinement predominantly in microvilli, as well as a lower ratio of NHE3 phosphorylated at serine-552 to total NHE3 (Pontes et al., 2015; Chen et al., 2017). In our study, higher NHE3 phosphorylation was observed in HLD rats, and treatments including SL, PL and CMT decreased the ratio of pNHE3/NHE3, suggesting that NHE3 might be one of the targets of the treatments we evaluated. Furthermore, the mucosal genus Phascolarctobacterium was positively correlated with pNHE3 and the ratio of pNHE3/NHE3. The treatments significantly decreased sMCT1 expression, which corresponded with a high concentration of luminal SCFAs and lower portal acetate and propiontate levels. Based on these findings, it is possible that SL and PL may improve disturbances in lactose metabolism by restoring an unbalanced intestinal bacteria and gut microbiota community structure.

Alterations in dietary carbohydrates have important effects on the composition and function of the gut microbiota. While the gut microbial community can be modified by dietary carbohydrates, it can also affect carbohydrates by playing a role in their metabolism (Gentile and Weir, 2018; Yadav et al., 2018). Many studies have shown that diverse types of dietary carbohydrates can also induce remarkable alterations in gut microbiota (Lopez-Legarrea et al., 2014; Yadav et al., 2018). Therefore, there is interaction between carbohydrates and the gut microbiota. In this study, PICRUSt analysis predicted that the gut luminal microbiota altered by HLD would show less fatty acid biosynthesis and metabolism, biosynthesis of amino acid, and more carbohydrate metabolism. SL treatment significantly upregulated fatty acid biosynthesis and metabolism and downregulated carbohydrate metabolism and membrane transport in the luminal microbiota, and decreased carbohydrate metabolism, translation and membrane transport while increasing fatty acid biosynthesis and metabolism and metabolic pathways in mucosa samples, suggesting that SL rectifies bacterial metabolism by modifying the gut microbial community structure. The results of functional prediction found SL and PL could restore the disorders in pathways of fatty acid, carbohydrate metabolism and ABC transporter caused by HLD, consistent with our previous results about higher SCFA concentration in luminal microbiota and musosal transporter alteration. CMT restored selected metabolic pathways compared to the model group, including upregulating biosynthesis of amino acids and secondary metabolites and downregulating carbohydrate metabolism and nucleotide metabolism in the luminal microbiota and increasing fatty acid biosynthesis and metabolism and decreasing carbohydrate metabolism and ABC transporter in the mucosal microbiota.

We transferred fecal microbes from SL- and PL-treated normal rats to non-treated diarrheal rats, which showed a significant delay in diarrheal onset and obvious remission of the severity of diarrhea. The results of 16S rRNA sequencing showed that the gut luminal and mucosal microbiotas were rejuvenated, with a significant increase in Akkermansia, Bifidobacterium, Ruminococcus gnavus, and Bilophila and a lower abundance of Lactobacilli and Dubosiella in the luminal microbiota, with a significant increase in Bilophila and Coriobacteriaceae_UCG-002, Escherichia-Shigella and a lower abundance of *Enterococcus*, *Helicobacter*, Dubosiella, and Collinsella in the mucosal microbiota.

In the present study, we want to investigate the effect of PL derived from SL in order to verify whether PL is one of an important effective constituent of SL. There are no differences in fecal water and cecal pH value after SL and PL treatment. However, PL showed a strong effect on food intake and weight gain than that of SL. Although the Sobs and Shannon indices was higher in SL treatment than that of PL treatment group, there is no difference in S-HLD and P-HLD. Both SL and PL could induce lactose fermentation and SCFA production. So we think PL play an important role in alleviating HLD-induced diarrhea, as an important effective constituent of SL.

**FIGURE 10:**
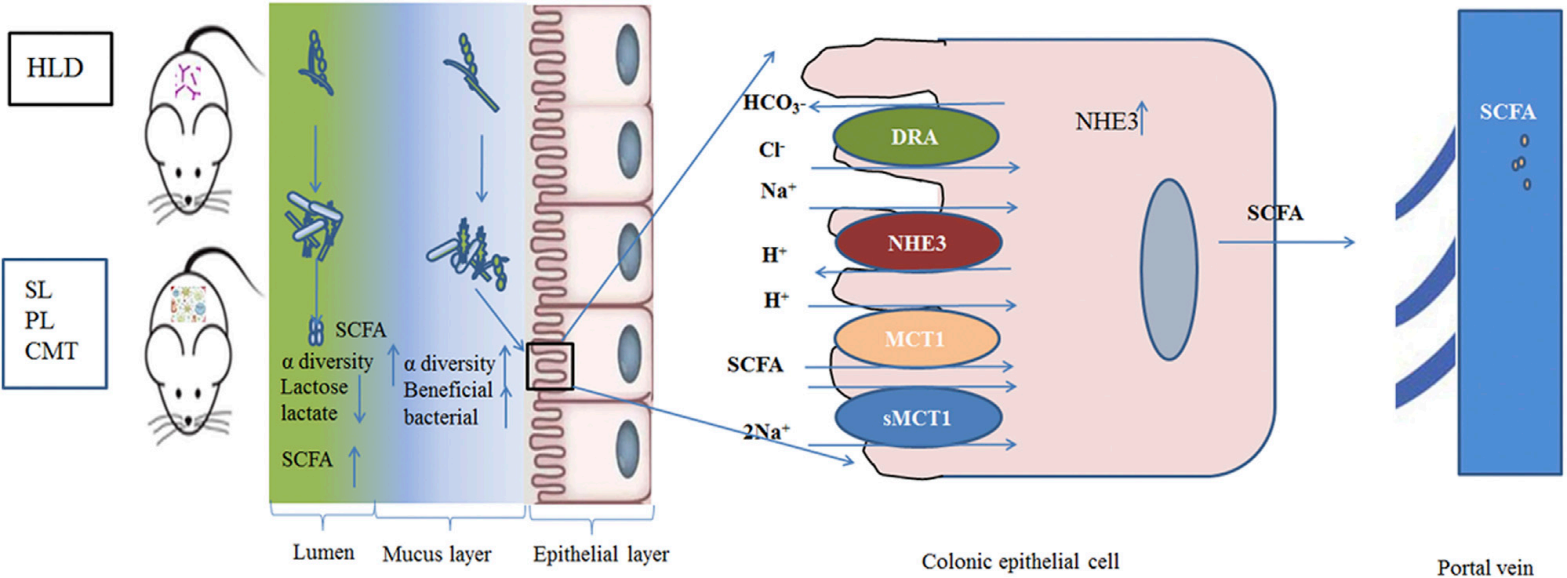
Schematic of the experimental proposition that SL, PL and CMT treatment positively modulate gut microbiota with associated enhancement of lactose fermentation in HLD rats.

In addition, SL and PL treatment effectively enhanced luminal lactose fermentation and SCFA production, which may be strongly associated with modulation of the gut luminal microbial community. A lower ratio of pNHE3/NHE3 and higher sMCT1 expression were found in the treatment group than in the model group, which may be closely related to the modulation of the mucosal microbial community. The regulatory mechanism partly contributed to the reshaping of the negatively impacted gut microbiota community by restoring their damaged metabolic pathways. This study demonstrated that SL and PL treatments alleviated the deterioration of high lactose-induced diarrheal symptoms, improved cecal lactose fermentation, regulated colonic luminal and mucosal microbiota, and restored intestinal ion transport (Figure 10).

Collectively, our findings demonstrate that SL and SL-derived crude polysaccharide had a positive therapeutic effect on HLD-induced diarrhea and its relationship with the luminal and mucosal microbiotas, which provides an important foundation for mechanism of SL action and developing PL-based treatment for lactose-induced diarrhea.

## Data Availability

The datasets presented in this study can be found in online repositories. The names of the repository/repositories and accession number(s) can be found below: https://www.ncbi.nlm.nih.gov; PRJNA809945.
