# Supplementary material for: Shen-Ling-Bai-Zhu-San (SL) and SL Derived-Polysaccharide (PL) Ameliorate the Severity of Diarrhea-Induced by High Lactose via Modification of Colonic Fermentation
Source: Front Pharmacol. 2022 Jun 28;13:883355. doi: 10.3389/fphar.2022.883355 (PMC9273845; doi:10.3389/fphar.2022.883355)
Supplement: Supplementary file 1 [file DataSheet1.docx]

Supplementary Material

## Supplementary Table


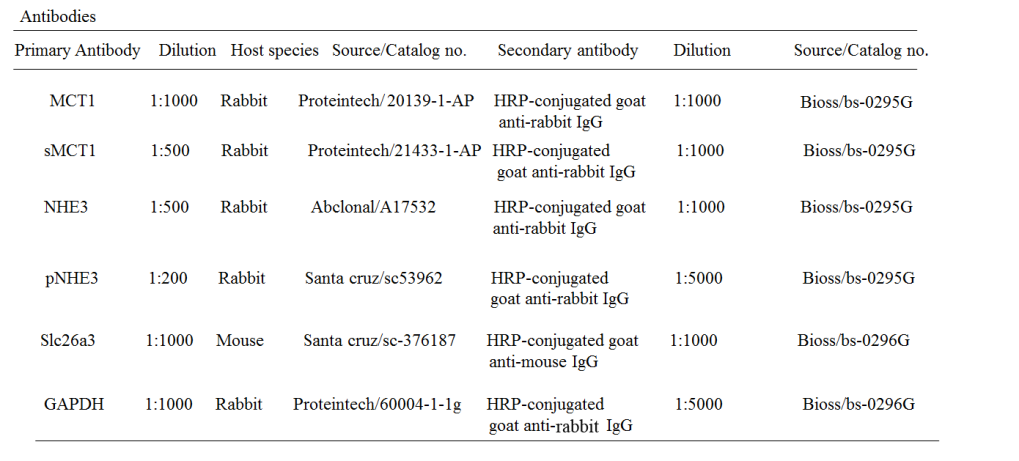


Supplementary Table Antibodies for western.

## Supplementary Figure


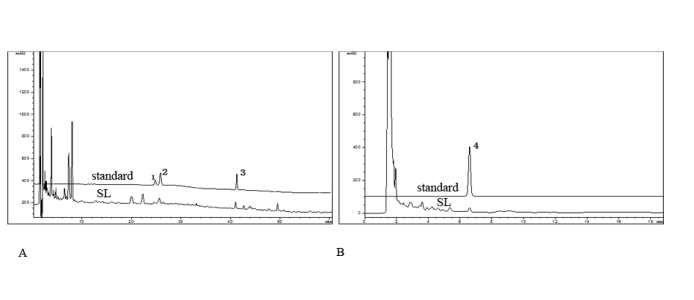


**Supplementary Figure 1** Chromatogram of Shen-ling-bai-zhu-san (SL) and standard chemical mixture analyzed by HPLC. 1. Ginsenoside Rg1; 2. Ginsenoside Re 3. Ginsenoside Rb1 4. Atractylenolide Ⅰ


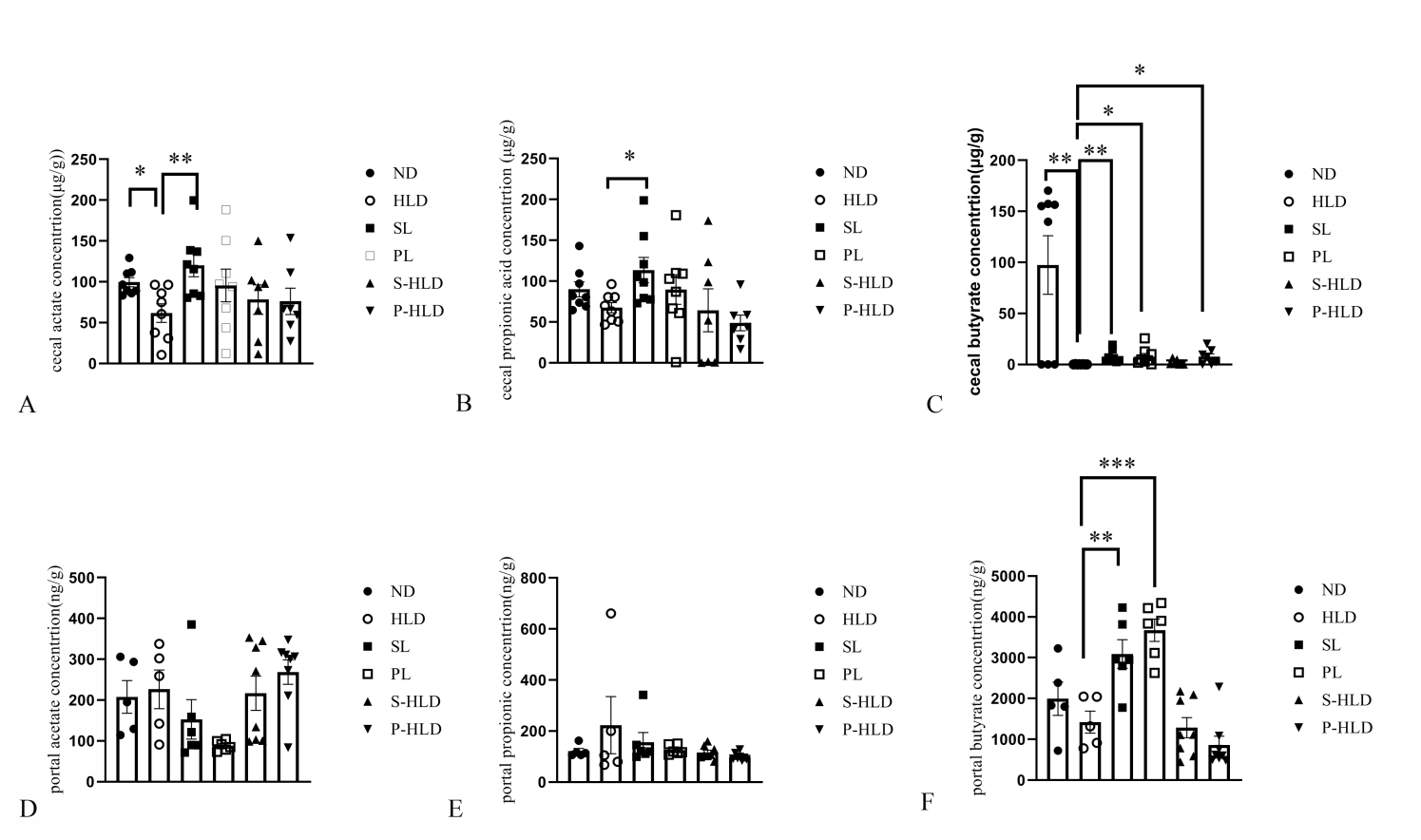


**Supplementary Figure 2** Concentrations of cecal and portal acetate (A, D), propionate (B, E), and butyrate (C, F) under different treatments.


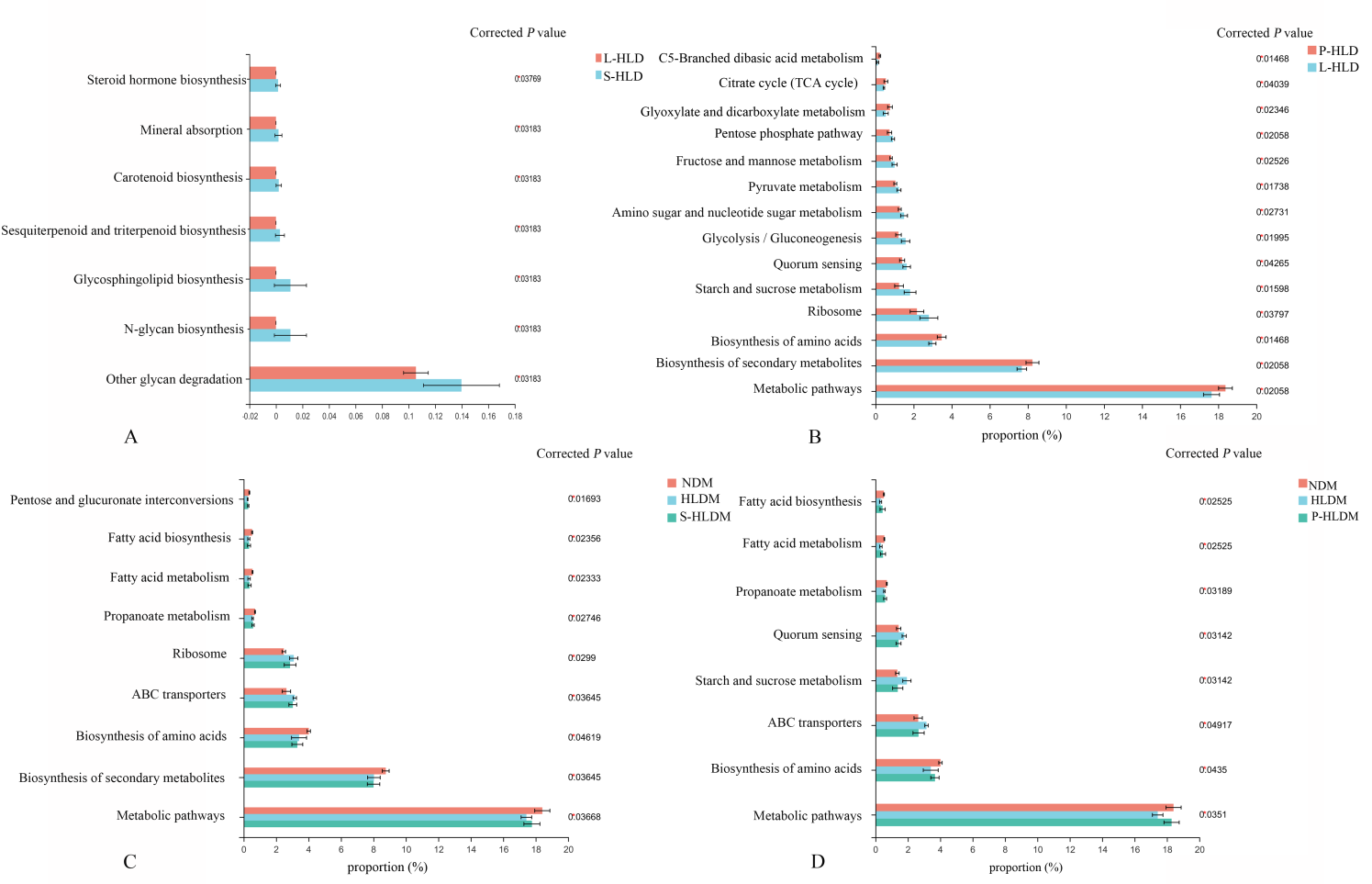


**Supplementary Figure 3** Functional prediction of altered luminal and mucosal microbiota based on KEGG pathways. (A) A total of seven significantly changed KEGG pathways in the luminal microbiota of the S-HLD group in comparison with that in the HLD group. (B) A total of fourteen significantly changed KEGG pathways in the luminal microbiota of the P-HLD group in comparison with that in the HLD group. (C) Few recovered KEGG pathways in the S-HLD group compared with the HLD group in mucosa microbiota. (D) A total of eighteen significantly recovered KEGG pathways in the mucosal microbiota of the P-HLD group.


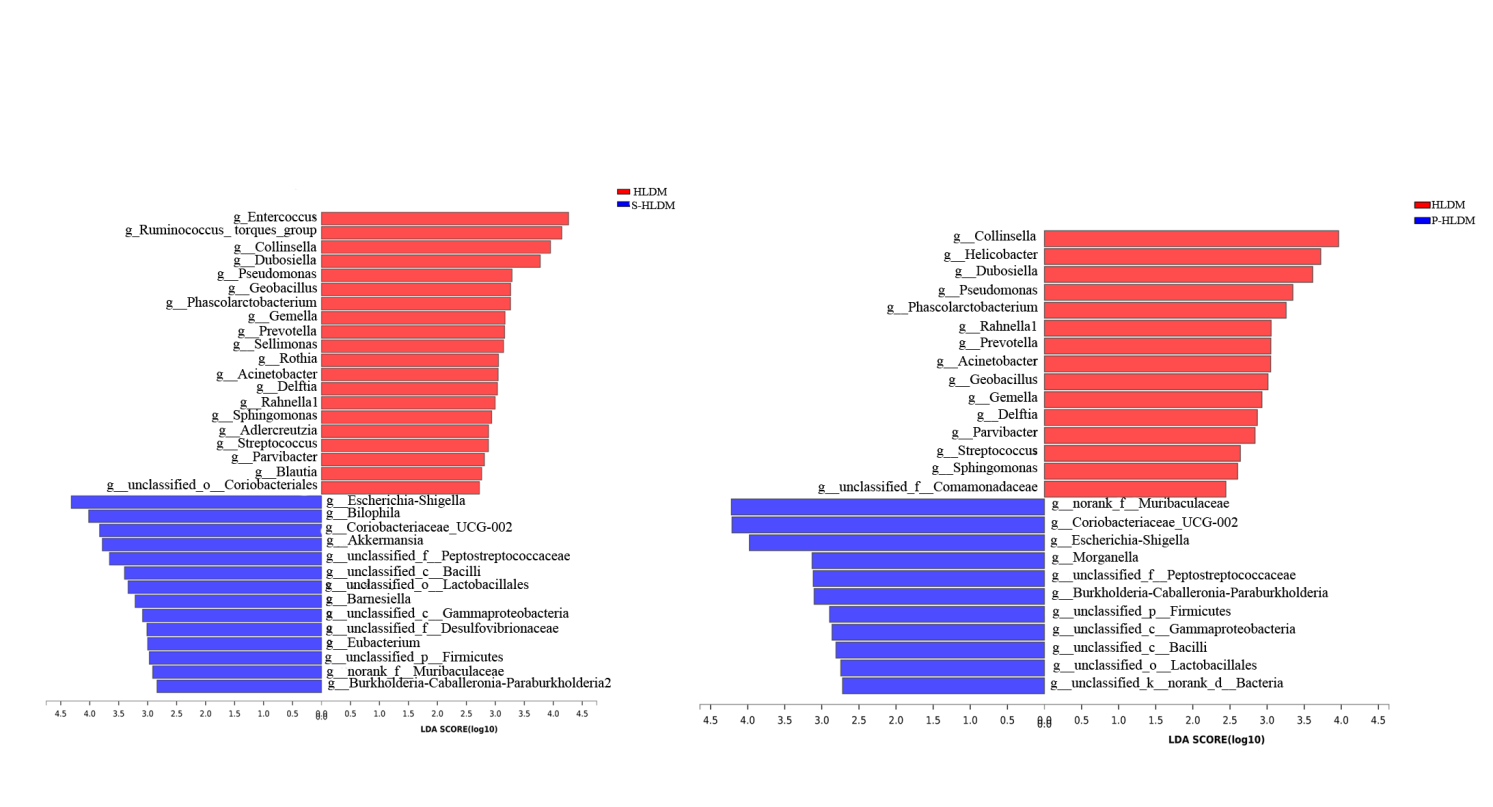


**Supplementary Figure 4** Linear discriminant analysis effect size (LEfSe) analysis of the mucosal microbiota differences between HLD& S-HLD (left) and HLD& P-HLD (right) at the genus level.
